# Maximizing the impact of malaria funding through allocative efficiency: using the right interventions in the right locations

**DOI:** 10.1186/s12936-017-2019-1

**Published:** 2017-09-12

**Authors:** Nick Scott, S. Azfar Hussain, Rowan Martin-Hughes, Freya J. I. Fowkes, Cliff C. Kerr, Ruth Pearson, David J. Kedziora, Madhura Killedar, Robyn M. Stuart, David P. Wilson

**Affiliations:** 1 Burnet Institute, 85 Commercial Rd, Melbourne, VIC 3004 Australia; 2 Department of Epidemiology and Preventive Medicine, Monash University, Clayton, Australia; 3 Centre for Epidemiology and Biostatistics, Melbourne School of Population and Global Health, The University of Melbourne, Melbourne, Australia; 4 Department of Infectious Diseases, Monash University, Melbourne, Australia; 5 Optima Consortium for Decision Science, Melbourne, Australia; 6 Department of Mathematical Sciences, University of Copenhagen, Copenhagen, Denmark

**Keywords:** Allocative efficiency, Budgeting, Malaria, Mathematical model, Nigeria, Optimization

## Abstract

**Background:**

The high burden of malaria and limited funding means there is a necessity to maximize the allocative efficiency of malaria control programmes. Quantitative tools are urgently needed to guide budget allocation decisions.

**Methods:**

A geospatial epidemic model was coupled with costing data and an optimization algorithm to estimate the optimal allocation of budgeted and projected funds across all malaria intervention approaches. Interventions included long-lasting insecticide-treated nets (LLINs), indoor residual spraying (IRS), intermittent presumptive treatment during pregnancy (IPTp), seasonal mass chemoprevention in children (SMC), larval source management (LSM), mass drug administration (MDA), and behavioural change communication (BCC). The model was applied to six geopolitical regions of Nigeria in isolation and also the nation as a whole to minimize incidence and malaria-attributable mortality.

**Results:**

Allocative efficiency gains could avert approximately 84,000 deaths or 15.7 million cases of malaria in Nigeria over 5 years. With an additional US$300 million available, approximately 134,000 deaths or 37.3 million cases of malaria could be prevented over 5 years. Priority funding should go to LLINs, IPTp and BCC programmes, and SMC should be expanded in seasonal areas. To minimize mortality, treatment expansion is critical and prioritized over some LLIN funding, while to minimize incidence, LLIN funding remained a priority. For areas with lower rainfall, LSM is prioritized over IRS but MDA is not recommended unless all other programmes are established.

**Conclusions:**

Substantial reductions in malaria morbidity and mortality can be made by optimal targeting of investments to the right malaria interventions in the right areas.

**Electronic supplementary material:**

The online version of this article (doi:10.1186/s12936-017-2019-1) contains supplementary material, which is available to authorized users.

## Background

Around 3.2 billion people worldwide are at risk of malaria. In 2015, there were 214 million clinical cases and 438,000 malaria-attributable deaths [1], the majority of which occurred in sub-Saharan Africa (88% of cases and 90% of deaths), making malaria a leading public health problem and economic burden for the region. Children under 5 years old are the most susceptible to infection, clinical disease and death, with 70% of all malaria deaths occurring among this age group [2].

A range of highly cost-effective interventions are available to reduce the burden of malaria. Vector control, including long-lasting insecticide-treated nets (LLINs), indoor residual spraying (IRS) and larval source management (LSM) have been demonstrated in multiple trials and in various settings to be highly effective in reducing infection and mortality [3–5]. Artemisinin-based combination therapy (ACT) is highly effective at treating clinical malaria and has contributed significantly to major reductions (~40%) in the global burden of malaria since being introduced in the 2000s [1]. Chemoprophylaxis has also been shown to be effective in high-risk populations: intermittent presumptive treatment during pregnancy (IPTp) can decrease still births, and seasonal mass chemoprevention in children (SMC) can reduce malaria-attributable mortality [6, 7]. However, limited funding to implement these interventions has resulted in substantial gaps in malaria control in many of the sub-Saharan African countries that still harbour substantial burdens of malaria.

Existing malaria modelling tools enable policy makers to estimate the epidemiological impact of scaling-up combinations of programmes or to estimate the requirements to achieve global targets [8–10]. However many countries, particularly in sub-Saharan Africa, are far from malaria elimination and require practical advice on how to allocate their current or projected budgets at state and country level in a way that achieves maximum impact. Presently there is a lack of quantitative tools to assist policy makers with these decisions. Allocative efficiency refers to the maximization of health outcomes using the most cost-effective mix of health interventions. ‘Optima’ is an analytic approach to assist decision-making around allocative efficiency. It was developed by the Burnet Institute and University of New South Wales in partnership with the World Bank to assist in optimizing resources for reducing the burden of diseases, particularly for HIV/AIDS [11, 12]. Optima has been used to successfully shift the allocation of actual budgets towards programmes with greater cost-effectiveness to improve health outcomes in over 40 countries [13–16]. Here, the Optima approach is applied to develop a model for malaria to specifically address this gap. The model can determine the optimal allocation of a given budget across a range of malaria interventions, geographical areas and risk populations, to minimize a user-defined objective (e.g., incidence, mortality).

In order to demonstrate the model’s capacity, an application is presented, conducted for the World Bank at their request, where the model was applied to the country of Nigeria. Nigeria accounts for more than 25% of the world’s malaria burden, more than any other country, with an estimated 59 million cases and 119,000 malaria-attributable deaths in 2013 [1]. Almost one in five deaths of Nigerian children under five were due to malaria [17], and malaria contributed to an estimated 11% of Nigerian maternal mortality [17]. Since 2009, a 10% reduction in incidence has been observed following large, donor-funded campaigns to distribute LLINs [1]. In addition, a series of performance-based contracts between the World Bank and the Nigerian Inter Faith Action Association (NIFAA) has resulted in increased utilization of programmes such as IPTp and LLINs [18]. However, 13 out of 37 Nigerian states have no reported funding for malaria control efforts, including four of the six poorest states in the northeast that have high malaria burdens (Adamawa, Taraba, Borno, Yobe [19–21]). Providing sustained funding to these areas is difficult due to the fragile political and security situation that limits access, and this lack of funding certainty means that despite the low cost and proven effectiveness of chemoprophylaxis for at-risk populations, SMC is only being implemented on a pilot basis and IPTp coverage remains low [19, 22, 23]. Optimizing the allocation of scarce funding in targeted geographical regions to maximize reductions in malaria morbidity and mortality is a priority for malaria control programmes in Nigeria and globally.

This paper describes an Optima Malaria model and demonstrates its use as a policy decision tool by presenting its application to Nigeria. Through a variety of donations from high-income countries and low-interest loans sourced and coordinated by the World Bank, US$300 million may be available in grant funds for malaria interventions in Nigeria over the next 5 years (2017–2022). The optimal allocation of current funding across malaria interventions, as well as the optimal allocation and impact of this additional funding, was assessed. This includes consideration of allocative efficiency across Nigeria’s six geopolitical regions, in order to demonstrate how shifting funding between regions can target resources to where they are most needed, producing even greater benefits at the country level.

## Methods

### Data synthesis to assess disease burden

Data on malaria incidence were obtained from the Malaria Atlas Project (MAP) [20, 21], and population and mortality data were obtained from the UN Population Division [24]. Details are provided in Additional file 1.

### Epidemic model

**Fig. 1:**
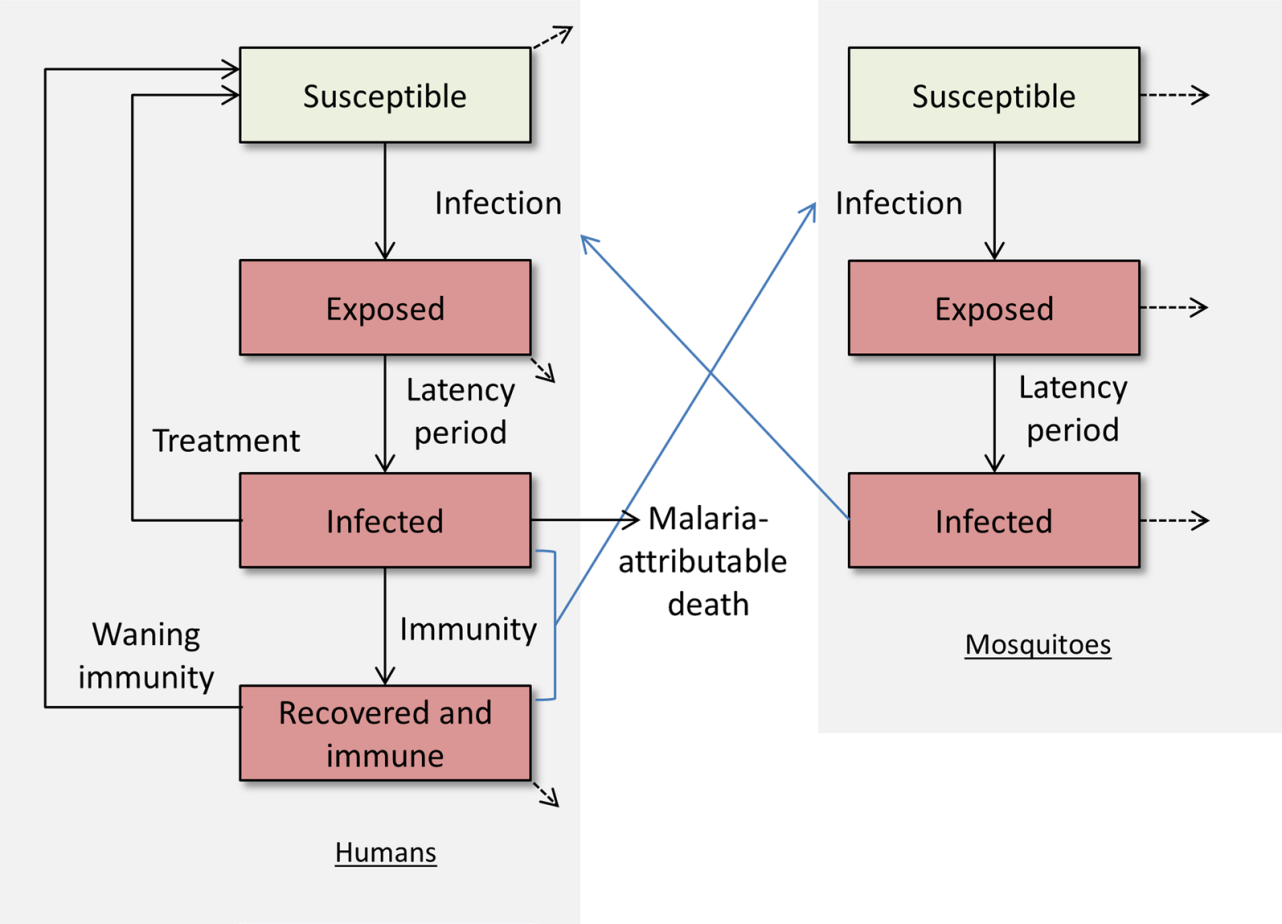
Model schematic. Compartments: susceptible, uninfected; exposed, infected with disease in the latent stage, approximating liver-stage infection; infected, infected with clinical symptoms approximating presence of circulating gametocytes and infectious to mosquitoes; recovered and immune, approximating clinically immune individuals who still have circulating gametocytes and are infectious to mosquitoes. The model is stratified by population group (children, defined as 0–5 years; pregnant women; and the rest of the population) and by the six geopolitical regions in Nigeria

The model contains a dynamic transmission model of *Plasmodium falciparum* (accounting for an estimated 85–100% of malaria cases in Nigeria [1, 25]) among humans and mosquitoes (Fig. 1). People in the model were distinguished as either: susceptible; infected with disease in the latent stage (approximating liver-stage infection); infected with clinical symptoms (approximating presence of circulating gametocytes and infectious to mosquitoes); or, recovered and immune (approximating individuals who have some exposure-acquired clinical immunity, but still have circulating gametocytes and are infectious to mosquitoes). The recovered and immune compartment was included in the model as the high rates of *P. falciparum* transmission in many countries means that exposure-acquired immunity is an important feature of the epidemic, and so this was accounted for by following the approaches of other modelling groups [8, 26–33]. When people in the model become infected they experience a latent period before becoming infectious, after which they either die or recover by either getting treated with drugs or developing clinical immunity. The model was stratified by population group (children, defined as 0–5 years; pregnant women; and the rest of the population, henceforth ‘general population’) and by each of the six geopolitical regions in Nigeria. Seasonality was included by scaling the total mosquito population size by a trigonometric function with period 1 year and amplitude depending on the extent of seasonal effects in each geographical region. Further details on the model structure, equations, calibration, parameters and their sources are provided in Additional file 1.

### Programmatic responses considered

Treatment of clinical malaria (i.e., people in the infected compartment) with ACT and seven intervention programmes were modelled: LLINs, IRS, IPTp, SMC, LSM, a behavioural change communication (BCC) programme and mass drug administration (MDA). The current coverage of each programme (by population group and region) was obtained from the National Malaria Elimination Programme (NMEP) End of Project Household Survey 2015 [23]; NMEP Malaria Key Indicator Survey 2015 [19]; SuNMaP Malaria Control State Fact Sheets [34]; and the Malaria Consortium [22]. Where no or limited estimates were available for specific regions or population groups, country estimates were used (see Additional file 1: Table C1).

For LLINs and IPTp, utilization was also considered. This was defined as the proportion of those with LLINs who slept under them, and the proportion of pregnant women who started IPTp that had the recommended three doses, respectively. These programmes were considered to be ineffective unless individuals were both covered and utilized them. Utilization could be increased by increasing the coverage of the BCC programme. Further details are provided in Additional file 1.

### Unit costs and effects of programmes

The unit costs and link between changes in programme coverage and model parameters were obtained from the literature and are summarized in Table 1, with sources and assumptions detailed in Additional file 1. As an example, LLINs have been shown to reduce the mosquito-biting rate by 56% for people who own and use them (Table 1, [8, 35]). If spending on LLINs is increased, the additional people in the model who become covered by the programme experience a reduction in mosquito bites, and therefore have a lower chance of becoming infected. This leads to a lower prevalence among humans, which in turn lowers the prevalence among mosquitoes and has flow-on effects even for people who were not covered by the LLIN programme.

**Table 1:** Estimated unit costs and effectiveness of malaria interventions in Nigeria

	Testing and treatment	LLIN	IRS	IPTp	SMC	LSM	MDA	BCC
Unit cost	Testing: $1.95 Treatment: child course = $0.65; adult course = $1.14	$2.61	$2.38	$1.10	$1.75	$1.65	$5.25	$0.03
Effectiveness reducing biting rate	–	56%	30%	–	–	–	–	–
Effectiveness killing mosquitoes	–	19%	56%	–	–	–	–	–
Effectiveness reducing mosquito density	–	–	–	–	–	52%	–	–
Effectiveness preventing new infections	–	–	–	95%	50%	–	90%	–
Effectiveness increasing utilization	–	–	–	–	–	–	–	20% LLINs; 30% IPTp.
Effectiveness clearing infections	95%	–	–	95%	95%	–	90%	–
Renewal time	As required	5 years	1 year	Per pregnancy	1 year	1 year	1 year	1 year
Assumed maximal achievable coverage with large resources	95% of infections	95%	95%	95% of pregnant women	Among children: 78% NW; 50% NE; 4% NC; 0% southern regions	25%	78% NW; 50% NE; 4% NC regions	95%

### Model calibration and validation

Data on annual incidence, number of malaria-attributable deaths, treatment numbers and prevalence were used to calibrate the model for each population group and region (Additional file 1: Table B3). This was done by calibrating parameters for the proportion who develop immunity following infection, the average duration of immunity, the malaria case fatality rate and the force of infection (the force of infection was dynamic and proportional to prevalence in the model, but the proportionality constant was varied) so that at equilibrium the model outcomes best fit available data for the 2015 incidence, mortality, treatment numbers and prevalence. The short malaria life cycle and rapid changes in response to intervention coverage over the previous 5 years [1] confounds any background trends in incidence and mortality, making it suitable to start a model from equilibrium for forward projections. However, the effects of changes in intervention coverage in the model on epidemiological outcomes still needed to be validated. The validation was performed by calibrating the model to 2010 epidemiological and programme coverage data (the only other year programme data were available, as malaria indicator surveys were only undertaken in 2010 and 2015 [19, 36]), and then linearly varying the coverage of programmes to 2015 values while running the model over this 5-year period. The resulting model estimates for 2015 incidence, prevalence and malaria-attributable deaths among each region and population group were then compared to 2015 data estimates.

### Optimization

The model was used to project an estimation of the total incidence of clinical malaria and malaria-attributable mortality over the next 5 years (2017–2022) associated with each allocation of funding (and corresponding programmatic coverage levels attained) between programmes, populations and regions. As the distribution of spending changes, so does the coverage of each programme in the model according to the unit costs in Table 1 with maximum coverage constrained due to logistical reasons. This varies the model parameters and leads to different values for total incidence and mortality. An adaptive stochastic descent optimization algorithm [37] was used to incrementally shift funding between programmes, population groups and regions in order to find the allocation that minimized: (1) incidence; and, (2) mortality. Two scenarios were considered for this purpose: (a) optimizing estimated current spending over the next 5 years (2017–2022); and, (b) optimizing estimated current spending with an additional US$300 million over the next 5 years (2017–2022). The optimal resource allocation among different interventions in each region was also simulated with incrementally less or more funding available. Further methodological details are provided in Additional file 1.

### Sensitivity analysis

Once the geospatially optimal allocation of funding was determined for each scenario, a multivariate sensitivity analysis was conducted to estimate bounds for the impact of allocating funding in this way (i.e., bounds for the number of deaths and cases prevented). Ninety-five per cent bounds were obtained for the number of deaths or cases averted using Monte Carlo sampling for the model’s structural parameters, unit costs and the effects of programmes, with samples taken from Normal distributions around point estimates (standard deviations of 5%, truncated at 10% above and below).

## Results

### Current burden of disease in Nigeria

**Fig. 2:**
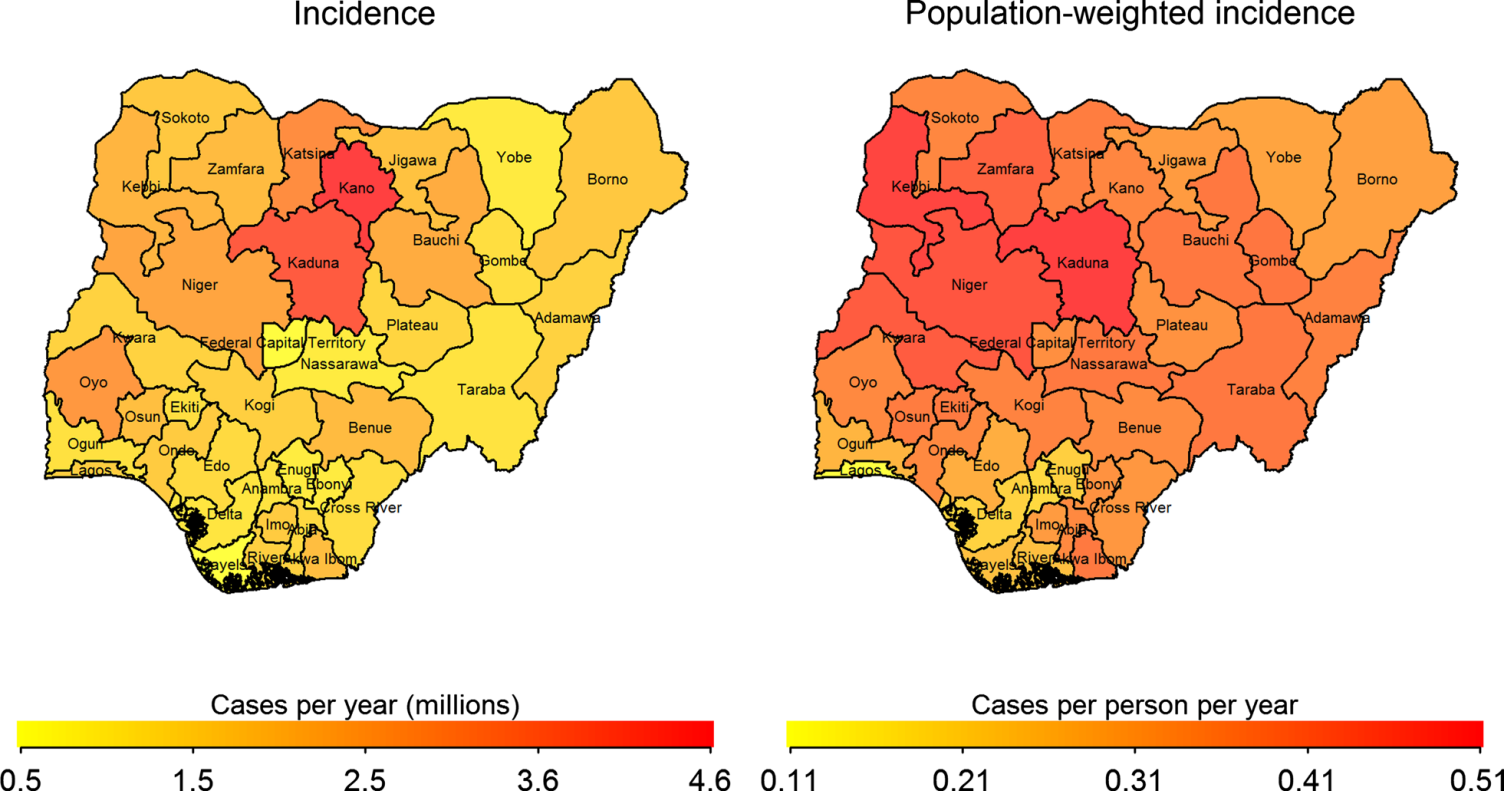
Annual malaria incidence and population-weighted incidence in Nigerian states (Source: Malaria Atlas Project [20, 24])

Current data indicate that the states with the largest proportion of malaria cases are Kano and Kaduna; however, on a per-capita basis, the North West and North Central regions have the greatest burden (Fig. 2; see Additional file 1: Appendix B for data sources). Current data also indicate that the per-capita rate of malaria-attributable mortality was the highest in the North Central and North West regions (Additional file 1: Figure D1). These data were used for model calibration.

### Current programmatic responses in Nigeria

Table 2 shows the current coverage of malaria prevention programmes in Nigeria, by region and population group. LLINs are the predominant preventive intervention, with the greatest coverage (86%) being in the North West region. Malaria is highly seasonal in this region, and is currently the only region to use SMC, albeit at low coverage (28% of children 0–5 years). Despite the malaria burden being the greatest in the northern regions, IPTp and IRS coverage were the lowest. In particular, IPTp coverage was less than half of all pregnant women for all regions except the South West.

**Table 2:** Estimated 2015 coverage of malaria interventions by population groups and geopolitical region. Sources: NMEP End of Project Household Survey 2015 [23]; NMEP Malaria Key Indicator Survey 2015 [19]; SuNMaP Malaria Control State Fact Sheets [35]; Malaria Consortium [22]

	NW (%)	NC (%)	NE (%)	SW (%)	SE (%)	SS (%)
2015 coverage						
IRS	5	1	3	1	7	5
IPTp (among pregnant women)	40	37	50	64	50	47
SMC (among children 0–5 years)	28	0	0	0	0	0
LLIN (among the general population)^a^	83	43	68	32	48	52
LLIN (among children 0–5 years)	94	65	82	57	73	71
LLIN (among pregnant women)	94	61	89	64	62	67
BCC [19]^b^	35	26	31	44	42	38
Utilization						
LLIN (among the general population)^c^[19, 23]	66	69	77	58	34	52
LLIN (among children 0–5 years)^d^ [19]	72	62	63	52	37	49
LLIN (among pregnant women) [19]	66	60	61	39	35	46
IPTp (among pregnant women)^e^[19, 39]	39	49	50	30	52	34

^a^LLIN coverage defined as the percentage of households with at least one net for every two people [37]

^b^Defined as the percentage reporting being exposed to prevention message

^c^Defined as the percentage of household members who slept under a mosquito net the previous night divided by the percentage of coverage

^d^Defined as the percentage of children under five who slept inside an LLIN last night among children in a household with at least one LLIN. This assumes that where an LLIN is available a child would preferentially use it over other household members

^e^Defined as the percentage of pregnant women who had at least three doses of IPTp among those who had at least one

Coverage of LLINs was greater among the two priority population groups (children 0–5 years and pregnant women) than for the general population (Table 2), and also where the burden of disease was the highest.

BCC programmes (typically operated through religious centres or targeted to healthcare workers) had greatest coverage in the southern regions (Table 2). Although these are not the areas where the malaria burden is greatest, these are the areas where utilization of LLINs is lowest, in particular among children [19, 23], suggesting the need for such programmes. In all areas, more than half of the population surveyed reported not being exposed to prevention messages [19], indicating the need to expand education and BCC programmes.

### Current spending on malaria programmes in Nigeria

Based on the unit costs of each malaria prevention or treatment programme (Table 1), the annual direct costs associated with the current coverage of programmes were estimated to be US$175,351,471 (see Additional file 1). By comparison, the World Malaria Report records that in 2014 the Nigerian Government reported direct malaria funding totalling US$285,931,583 (entirely donor-funded: Global Fund US$137,920,815; the World Bank US$52,220,588; PMI/USAID US$73,771,000; other bilaterals US$20,157,565; WHO US$861,615; and UNICEF US$1,000,000) [1]. There are several key factors that may explain the difference between this estimate and the 2014 value from the World Malaria Report. Firstly, this estimate does not include the costs of non-direct programmes such as central management and surveillance. Second, the cost of achieving this level of LLIN coverage was assumed to be spread evenly over the past 5 years (given their 5-year lifespan). If the majority of currently owned LLINs were purchased in more recent years, then the expenditure in these years would be considerably higher. Third, given the funding for malaria in Nigeria is entirely from donors, there is likely to be substantial variability between years.

**Fig. 3:**
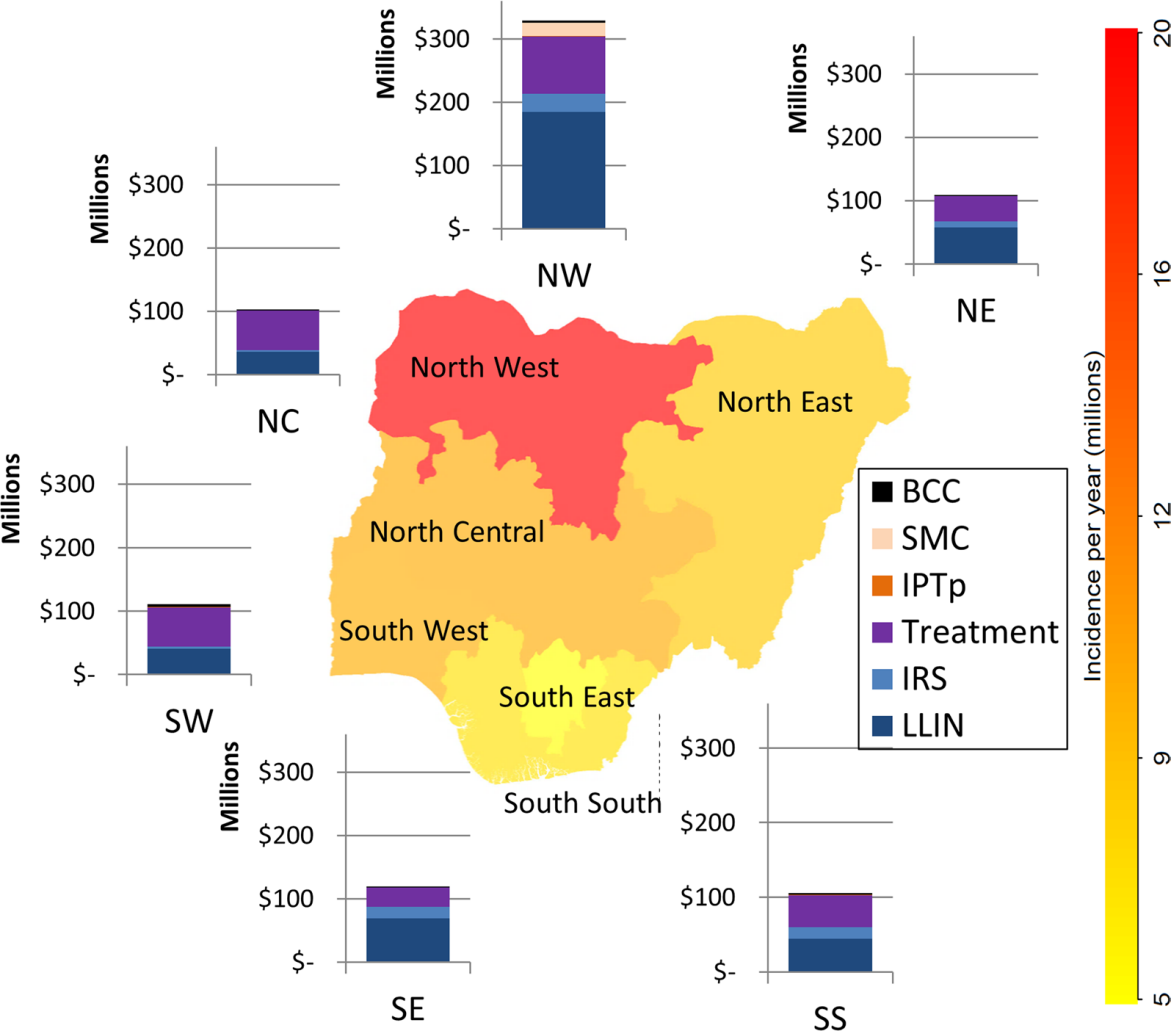
Estimated current annual spending by region and programme, according to 2015 coverage. Values for each population group and sources are provided in Additional file 1

The programme receiving the greatest amount of funding was LLINs, followed by treatment (including testing costs), while the North West region was the one receiving the most current funding for programmes (Fig. 3).

### Model calibration and validation

After calibrating model parameters, model outcomes for annual incidence, mortality and treatment numbers fit the 2015 data in each region and among each population group well (Additional file 1: Figure A2).

**Fig. 4:**
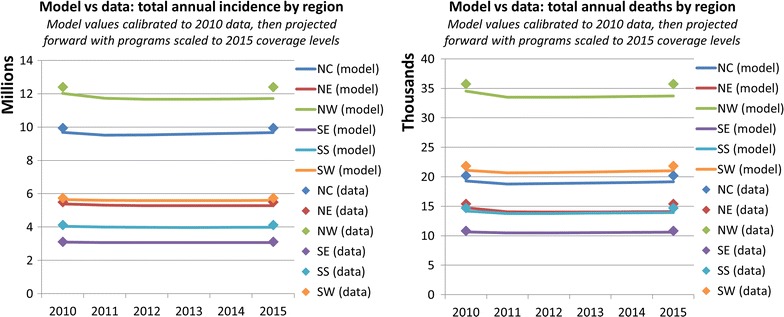
Model validation exercise showing the effects of changes in programme coverage in the model on epidemiological outcomes. Programme coverage data were available for 2010 and 2015 [19, 37]. The model was calibrated to epidemiological and programme coverage data from 2010 and then projected forward by linearly varying the coverage of programmes to 2015 values

Results of the model validation exercise are shown in Fig. 4. The model also calibrated to 2010 data well. Projecting the model to 2015 with programme coverages changing from 2010 to 2015 levels only produced marginal changes in the total annual incidence and deaths in each region; however this was consistent with the minimal variations observed in the data over this period. It is possible that any discrepancies could be due to the greater uncertainty in the coverage levels of programmes in 2010; in particular there were no data to reflect coverage of any BCC programme, which was therefore assumed to be zero, despite the possible existence of undocumented education campaigns.

### Region specific optimization

**Fig. 5:**
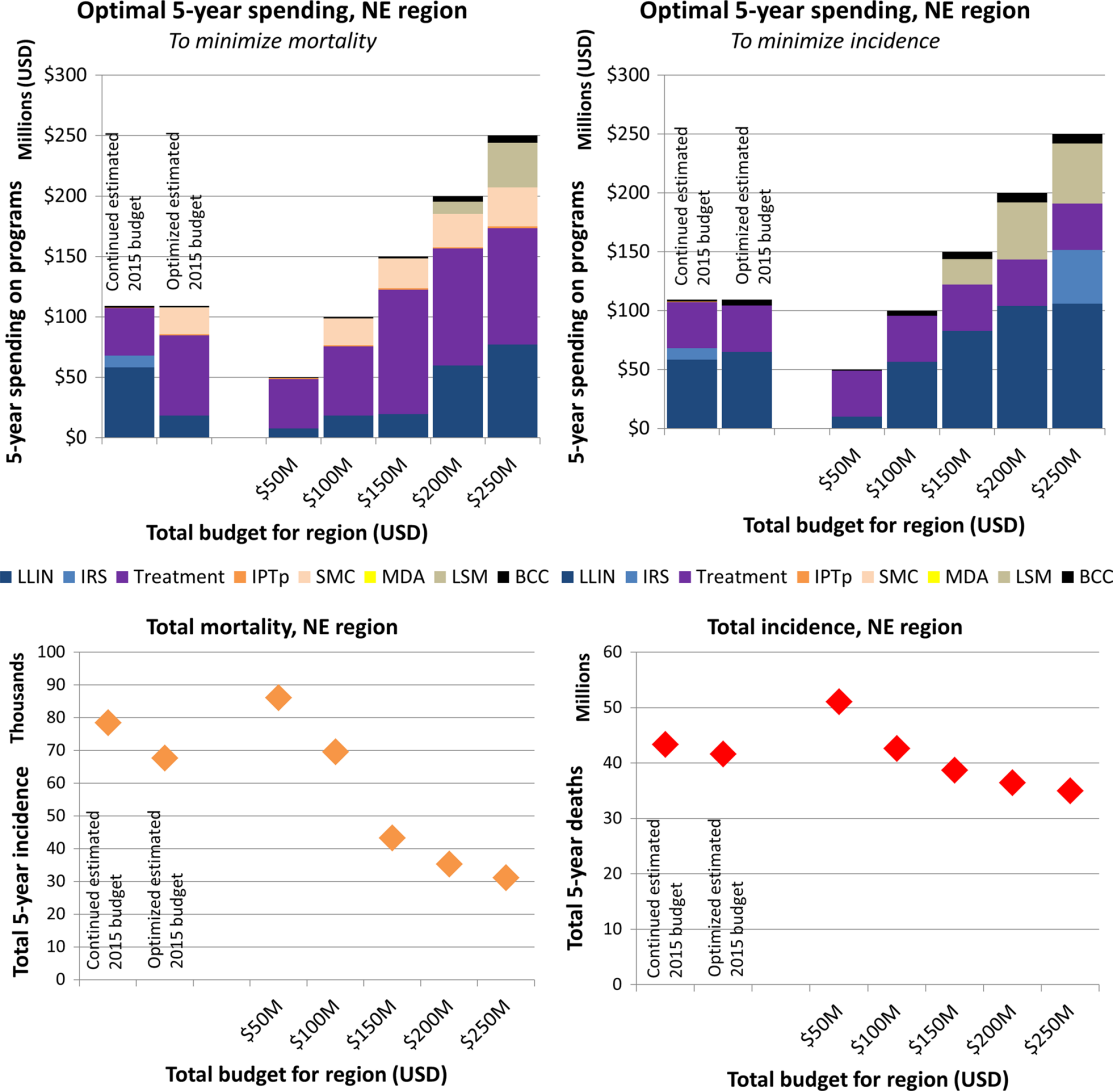
Estimated current and optimal 5-year spending allocations on programmes in the North East (NE) region for varying total budget levels. Optimized to minimize malaria-attributable mortality (left) or incidence (right)

The optimal allocation of funding within each region varied depending on whether the objective was to minimize mortality or incidence, and also as total available budget was increased (Fig. 5 for the North East region, Additional file 1: Figures D2–D7 for other regions).

In general, when optimization was performed within each region the results suggested that treatment and LLINs should be funded as a priority intervention and that IPTp and BCC are cheap and effective and should also be funded. Furthermore, when the objective was to minimize mortality, treatment expansion combined with SMC in seasonal areas is deemed to be critical and prioritized over LLIN, while LLIN remained a priority when minimizing for incidence. For appropriate areas with low rainfall, LSM is prioritized over IRS due to lower cost and comparable effectiveness, whereas MDA is not recommended unless other programmes are established (Additional file 1).

### Geospatial optimization

The optimal geospatial allocation of funding was similar regardless of whether the objective was to minimize mortality or incidence over the next 5-year period, with the exception that the South West region was prioritized over the South region for funding to minimize mortality and vice versa to minimize incidence. Within each region the actual programmes being funded varied under different scenarios (consistent with Fig. 5).

**Fig. 6:**
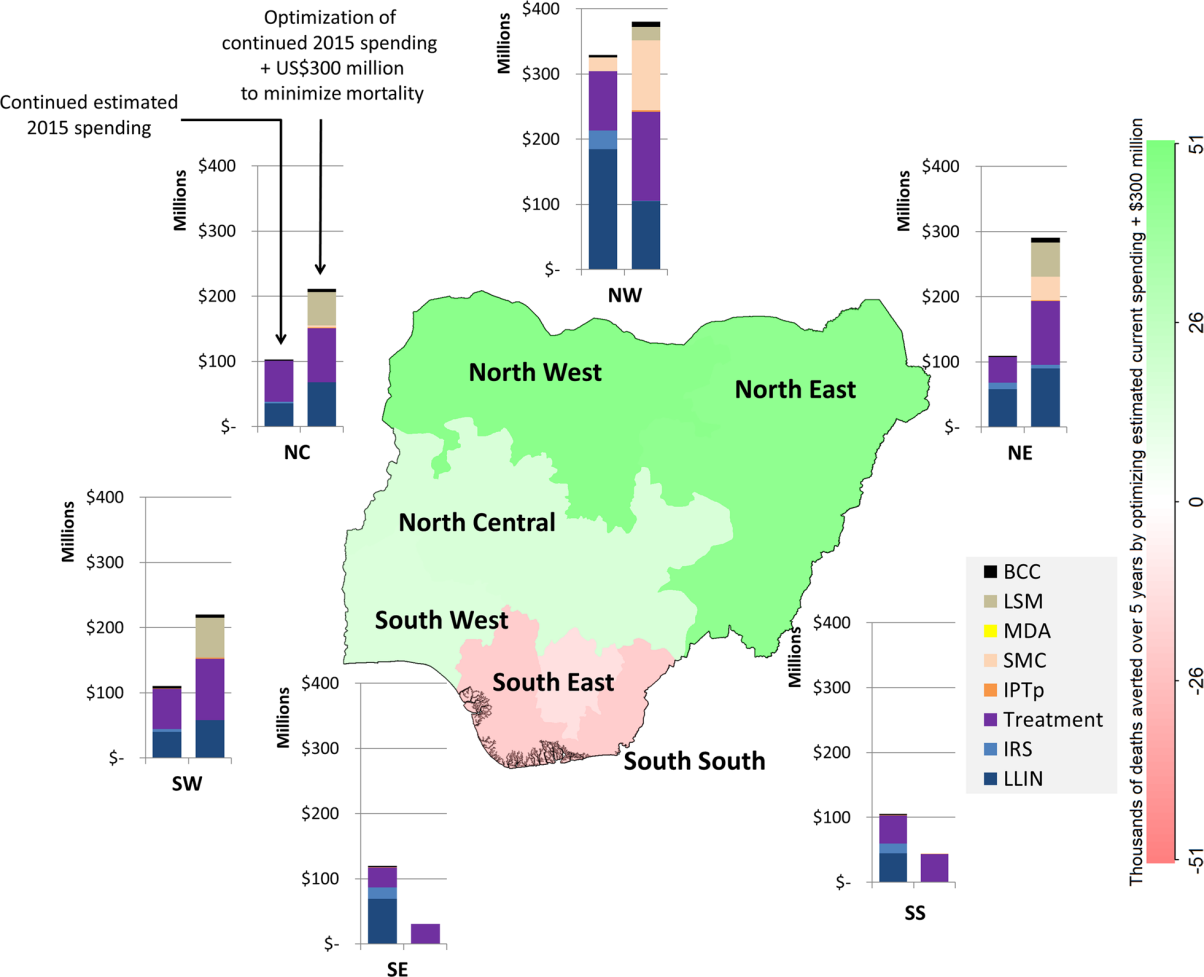
Geospatial optimization to minimize mortality. Optimized 5-year spending of estimated current budget +US$300 million allocations compared to estimated current (non-optimized) spending

The model estimated that a total 83,611 (76,022–106,712) deaths (15%), could be averted over 5 years by optimizing the estimated 2015 spending (Additional file 1: Figure D10), and 134,384 (114,883–142,858) deaths (24%) could be averted over 5 years by optimizing the estimated 2015 spending +US$300 million to minimize mortality (Fig. 6).

**Fig. 7:**
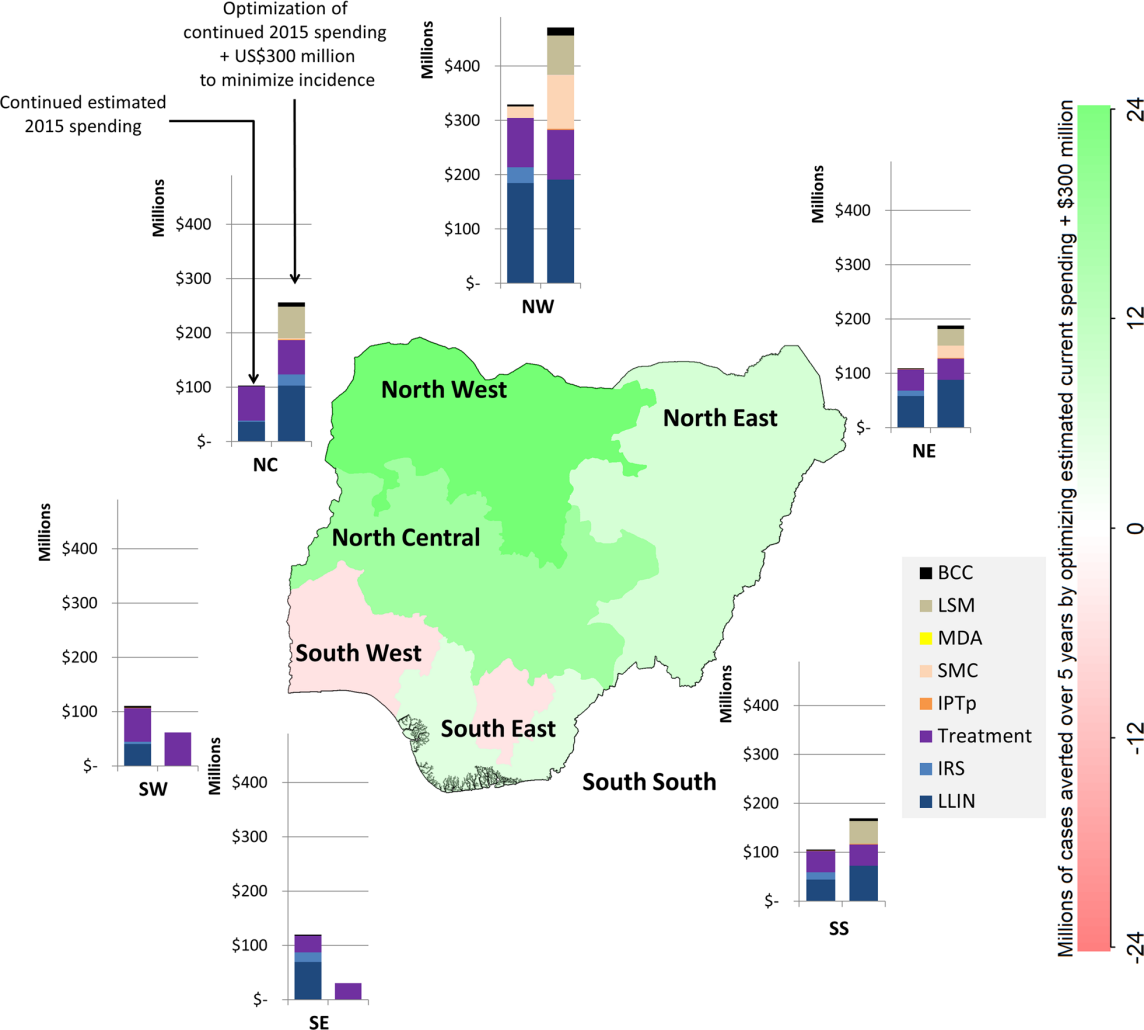
Geospatial optimization to minimize incidence. Geospatially optimized 5-year spending of estimated current budget +US$300 million allocations compared to estimated current (non-optimized) spending

The model estimated that a total 15.7 (13.7–23.3) million cases (5%) could be averted over 5 years by optimizing estimated 2015 spending (Additional file 1: Figure D11), and 37.3 (34.0–47.8) million cases (11%) could be averted over 5 years by optimizing estimated 2015 spending +US$300 million (Fig. 7).

To minimize country-level mortality or incidence over the 5-year period the model ultimately suggests that the largest shifts in funding should be from the southern regions to the northern regions. Despite leading to an overall reduction in mortality or incidence, this shift in funds resulted in slightly worse outcomes for some of the southern regions.

**Fig. 8:**
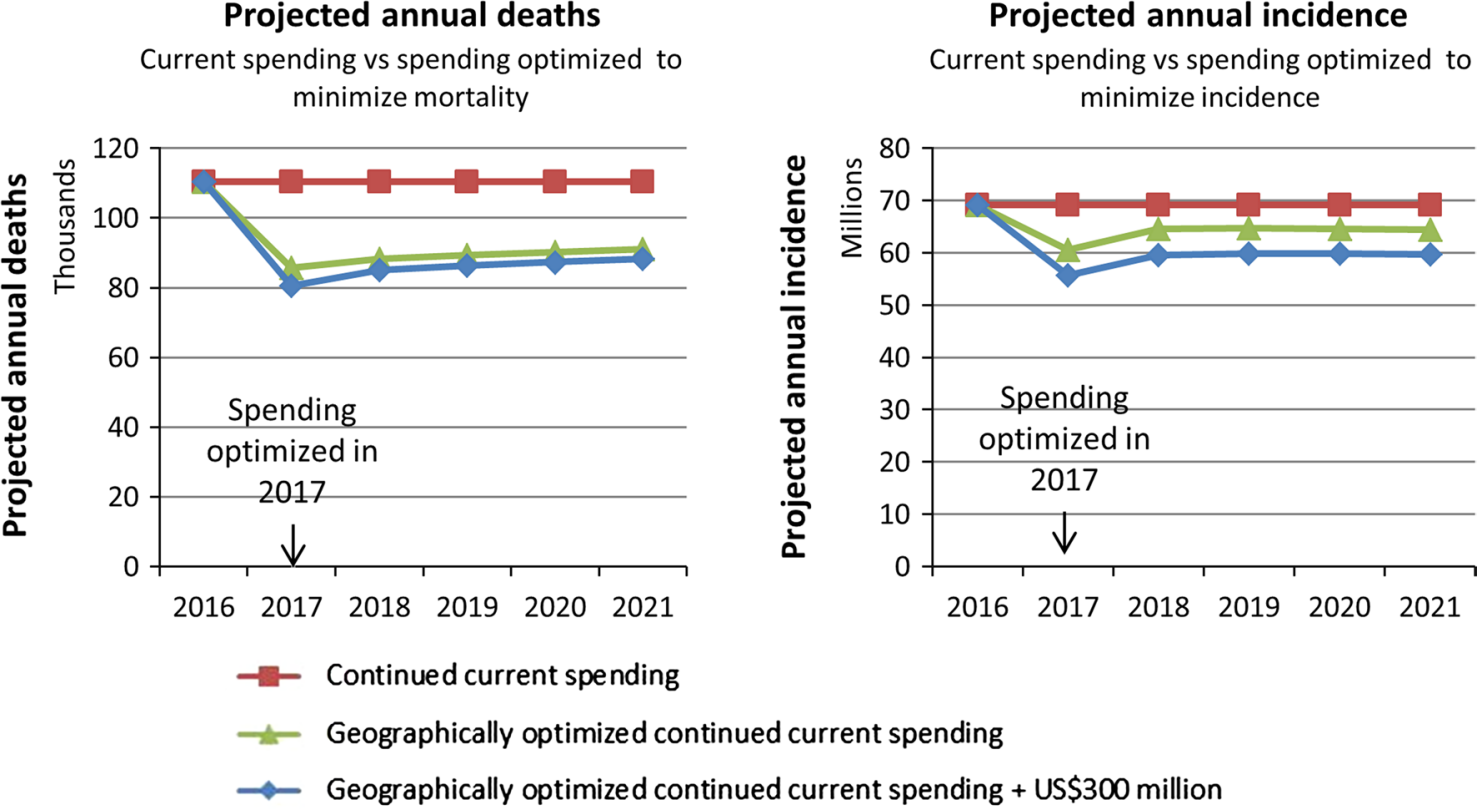
Model projections for annual malaria incidence and malaria-attributable deaths. Left: annual malaria-attributable deaths with continued current spending, continued current spending optimized to minimize mortality, and continued current spending +US$300 million optimized to minimize mortality. Right: annual malaria incidence with continued current spending, continued current spending optimized to minimized incidence, and continued current spending +US$300 million optimized to minimize incidence

Although geographically optimizing spending led to substantial reductions in malaria-attributable deaths and malaria incidence, even with an additional US$300 million there were still nearly 90,000 deaths and 60 million cases of malaria each year (Fig. 8).

## Discussion

A geospatial, epidemic model coupled with an economic and costing framework was developed to estimate the optimal allocation of funding across population groups and programmes in order to minimize malaria incidence or malaria-attributable mortality. When applied across geopolitical regions of Nigeria the model predicted that allocative efficiency gains could avert approximately 84,000 (15% of) deaths or 15.7 million (5% of) malaria cases over 5 years, if the estimated current funds (approximately US$175 million per annum) for malaria control continued to be available. If an additional US$300 million were available for this 5-year period, then optimal spending could avert approximately 134,000 deaths (24% of) deaths or 37.3 million (11% of) malaria cases. Although these are substantial gains, even with this significant additional funding there were still nearly 90,000 deaths and 60 million cases of malaria each year, supporting previous modelling that indicates the current range of malaria interventions may be inadequate to address the epidemic in high-prevalence countries [8].

Geospatial analysis results suggest that additional reductions in incidence and mortality are possible by focussing funding on areas that have the highest burden of malaria. In the example of Nigeria, this is the northern regions, although in practice this may be difficult for the Nigerian Government due to conflict, war and other security threats occurring in these areas. More generally this analysis demonstrates that by increasing granularity, interventions can be more accurately targeted to generate larger allocative efficiency gains. It was possible to assess this in Nigeria due to the geo-specific data and parameter estimates obtained from the National Malaria Elimination Programme surveys [19, 23], but as countries move towards malaria elimination the increasing resolution required to inform optimal resource allocation will rely far more heavily on surveillance systems within countries. There is therefore a need to ensure data capture and monitoring systems are established to enable this to occur.

Consistent with the literature, LLINs were found to be critical for preventing malaria and treatments critical for preventing mortality [8]. Once treatment, LLINs, IPTp, and BCC programmes had reached sufficient coverage then the model suggested that SMC should receive priority funding to reduce mortality and LSM should receive priority funding to reduce incidence. The different optimal allocations when minimizing either incidence or mortality highlights the need for clear and specific strategic targets, designed in consultation with country representatives, implementation partners, community organizations, and other stakeholders. Once these have been established, many conceivable objective functions can be used within the model’s optimization procedure to determine how resources should be optimally allocated. For example, possible candidates include minimizing disability-adjusted life years (DALYs), which could be implemented as a weighted sum of incidence and mortality over each population group, minimizing deaths whilst ensuring that no regions become worse off, or ensuring high coverage of particular interventions among children before any other programmes are funded. Further, Nigeria had a specific need to optimize over the next 5-year period; however countries will need to define the time period of their strategies because optimizing over different time frames can change priorities. For example, lowering incidence in the short term can lead to a lower prevalence and reduce deaths in the longer term.

Once a strategic goal is defined, the feasibility of shifting current funding allocations towards a more optimal mix must be considered. A potential political implementation challenge could be that to maximize the overall impact of funding some geographical areas would be worse off. However, in many resource-limited countries (including Nigeria) malaria programmes receive large amounts of donor funding [1], which may be less likely to be tied to particular programmes and areas than government-funded programmes. When presented with quantitative evidence of overall better outcomes, donor funding supporting malaria elimination programmes may have the flexibility to make these changes.

This analysis found LSM to be prioritized over IRS and that it was therefore more cost-effective when combined with other malaria programmes; however, it must be emphasized that there is well-established evidence supporting the effectiveness of IRS, whereas the evidence to support the effectiveness of LSM is much weaker [4, 5]. Therefore, this finding must be considered in the specific context of Nigeria, where prior infrastructure investment in LSM has occurred over the past 5 years [38, 39]. In this context, these results support the continued introduction and scale-up of LSM programmes in Nigeria subject to rigorous local analysis of environmental suitability in terms of the number, type and accessibility of water sources. At this stage, the limited number of studies on the effectiveness of LSM is insufficient to support the general recommendation of a movement away from the status quo of IRS programmes to develop LSM capacity in other settings.

The approach used in this paper is able to produce practical outputs to inform policy. The findings also demonstrate that allocative efficiency analysis can go beyond the scope of cost-effectiveness league tables; by considering the optimal allocation of a series of increasing budgets for each region, the model determined that additional interventions may be added to the optimal mix before the current interventions have reached saturation coverage.

The model is flexible enough to incorporate additional features that are likely to influence outcomes where data are available. For example, the approach taken allows for the cost and effectiveness of programmes to vary by region. This means that if evidence is found of the effectiveness of programmes decreasing due to widespread drug resistance or insecticide resistance, it can be included in future analyses, and that the cost of different delivery modes for programmes can be included for settings where (unlike Nigeria) programmes are not delivered through private sector contracts. This could also include updates incorporating the partial utilization of programmes: the binary measure of IPTp utilization may understate the actual effectiveness of the programme, since pregnant women who receive fewer than three doses of IPTp may experience partial protection. However, even with potentially underestimated effectiveness, IPTp received priority funding.

Population groups and their movements can also be varied. In particular, as malaria is endemic across Nigeria and the burden is high relative to neighbouring countries, immigration, movements between regions and ‘importation’ of malaria infections were not modelled but could be included in other settings. A single, density-dependent population of mosquitoes was also assumed for each region; however, differences in mosquito population characteristics can be varied to account for differences in climate and species between regions. Although annual average costs were used for seasonal-dependent programmes, the use of annual averages is suitable for budgetary purposes if seasonality is considered to be an implementation issue: for example, IRS should be implemented before the peak season begins. For programmes such as SMC, unit costs were reduced to account for the intervention only being required for parts of the year.

There are limitations in knowing what proportion of funding commitment could actually be translated into end product. This issue is minimized in Nigeria, as unit costs came largely from private sector reports where a specified number of units were delivered for a total contract value, e.g., [18, 34], thus accounting for funding being lost to inefficiencies or being otherwise unaccounted for. Finally, as with all mathematical models, there is uncertainty in each parameter. In particular there is a lack of randomized intervention trials to inform setting-specific effectiveness assumptions for each programme. Even though some of this uncertainty was accounted for by including confidence bounds, there remains inherent uncertainty associated with the choice of model structure. The recovered compartment represents infected individuals who have exposure-acquired clinical immunity but who are still able to transmit parasites to the mosquito population. Immunity acquisition is known to be age-dependent, which was implicitly modelled by separating the effects of immunity among children (the likelihood of developing immunity, the duration of immunity and the risk of death per infection) from the rest of the population. Immunity was also modelled to wane based on the background entomological inoculation rate (following [26, 30, 40], Additional file 1). This approach is pragmatic given the limited geographically specific data on immunity available; however there are many modelling groups investigating the effects of immunity with different model types and model structures (including within-host models, age-structured models, partial differential equations models with immunity as a continuum and explicitly implementing different types of immunity such as immunity at the sporozoite and liver stages and blood-stage immunity [26–29, 41–43]). Future model expansions could consider how to best structure a population-level compartmental model to approximate these complex effects in the context of available data.

## Conclusions

The use of quantitative tools to guide malaria budget allocation decisions can save a significant number of lives. In Nigeria, allocative efficiency gains could avert approximately 84,000 (15% of) deaths or 15.7 million (5% of) malaria cases over 5 years, if the estimated current funds for malaria control continued to be available. This requires tailoring programme focus areas for specific regions and targeting funding to places that it can have the greatest impact.

## Additional file

**Additional file 1.** Detailed description of epidemiological model (including equations, program implementations and calibration), model parameters, estimates of current spending and additional charts.

## Abbreviations

BCC: behavioural change communication
IPTp: intermittent presumptive treatment during pregnancy
IRS: indoor residual spraying
LLIN: long-lasting insecticide-treated net
LSM: larval source management
MDA: mass drug administration
SMC: seasonal mass chemoprevention in children
