# Supplementary material for: Maximizing the impact of malaria funding through allocative efficiency: using the right interventions in the right locations
Source: Malar J. 2017 Sep 12;16:368. doi: 10.1186/s12936-017-2019-1 (PMC5596957; doi:10.1186/s12936-017-2019-1)
Supplement: Supplementary file 1 — Additional file 1. Detailed description of epidemiological model (including equations, program implementations and calibration), model parameters, estimates of current spending and additional charts. [file 12936_2017_2019_MOESM1_ESM.pdf]

Supplementary material

Maximizing the impact of malaria funding through allocative efficiency: using the right interventions in the right locations

Appendix A: Detailed description of epidemiological model

This appendix provides a mathematical description of the model used for the analysis. A schematic is shown in Figure A1.

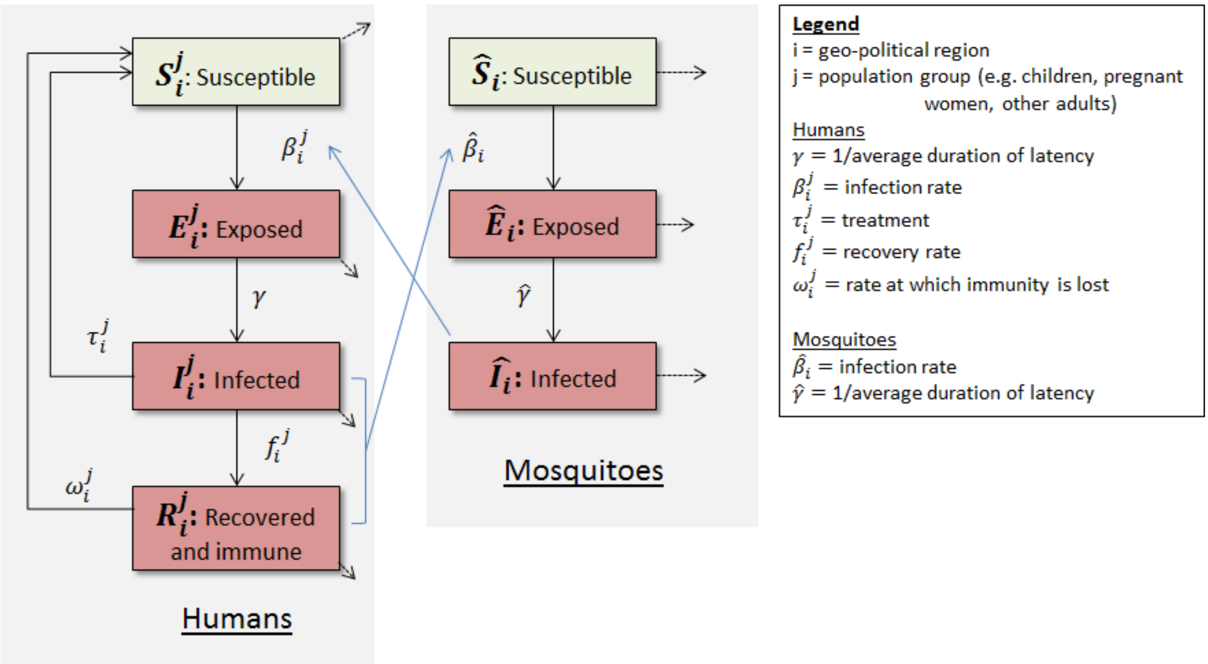

Figure A1: Model schematic.

Population and compartments

The compartments and indices are defined as:

- Human compartments: S, susceptible; E, exposed (liver-stage); I, infectious (blood stage); R, recovered (temporarily immune);
- Mosquito compartments: $\hat{S}$, susceptible; $\hat{E}$, exposed; $\hat{I}$, infectious;
- Subscript i represents the different geopolitical regions in the setting of the application;
- Superscript j represent the different population groups (0-5 year old children, pregnant women, other).

For humans, deaths are assumed to occur at equal rates from the S, E and R compartments (equal to the all-cause mortality rates for that population group), but with an increased rate from the I

compartment. Mosquito mortality is assumed to be constant in the absence of specific interventions or environmental changes. Total human population sizes and growth rates were taken from UN population data [1, 2] for each population group and region, and population growth was assumed to occur in the susceptible compartment. Explicitly, these are given by:

- μ^j = human mortality rate for population j in the S, E and R compartments
- $\mu_i^j + \Gamma_i^j$ = human mortality rate for population j in the I compartment (i.e., mortality due to infection is increased by an amount Γ_i^j)
- $\overline{\overline{\mu^j}}$ = growth rate for population j

Mosquito population sizes were maintained by assuming birth rates were equal to death rates; however the total population size was scaled between seasons depending on a seasonality factor:

- $\hat{\mu}$ = mosquito mortality rate from each compartment [1 / life expectancy]. It is also assumed that mosquitoes are born into the $\widehat{S_i}$ compartments at the same rate to maintain a constant population.
- $\widehat{pop} = constant * \left(1 + \kappa_i \sin\left(\frac{2\pi t}{365}\right)\right)$ is the total mosquito population size, where κ_i is a seasonality constant for region i. κ_i represents the difference in mosquito population density between the wet and dry seasons.

For the northern regions of Nigeria, the entomological inoculation rate (infective bites per person per night) was estimated to be 0.37 in the dry season, 1.24 in the rainy season compared to an annual average of 0.83 [3]. Therefore κ_i was calibrated to ensure that in the northern regions:

(peak_incidence – min_incidence) / average incidence = $(1.24 - 0.37)/0.83 = 1.05$

while κ_i was set to be zero in the southern regions based on considerably smaller seasonal effects [4].

Transmission

Transmission is defined in terms of bites per month, probability of transmission per bite, and the current infectious proportion of the population. Let

- N_i^j = bites per month among population group j in region i (calibrated to malaria incidence among population group in each region)
- $\widehat{N_i}$ = bites per month in mosquito population for region i, where the total bites among all humans balances the total bites among mosquitoes:

$$\widehat{N_i} = \frac{\left(\sum_j N_i^j * pop_i{}^j\right)}{mosquito\ pop_i}$$

- λ = probability of transmission [M→H] per bite
- $\hat{\lambda}$ = probability of transmission [H→M] per bite

Then in region i, and among population group j, the forces of infection for humans (β_i^j) and mosquitoes ($\hat{\beta}_i$) are given by

$$\beta_i^j = \mathrm{N}_\mathrm{i}^j\lambda * \frac{\hat{I}_i}{\widehat{S}_\iota + \hat{E}_i + \hat{I}_i}$$

$$\hat{\beta}_i = \widehat{\mathrm{N}_\mathrm{i}}\hat{\lambda} * \frac{\sum_j(I_i^j + R_i^j)}{\sum_j(S_i^j + E_i^j + I_i^j + R_i^j)}$$

Other parameters

Movements occur between compartments at the following rates:

- $\gamma = 1$ / average duration of latency in humans [time from bite to infectious];
- $\hat{\gamma} = 1$ / average duration of latency in mosquitoes [time from bite to infectious];
- $f_i^j =$ recovery rate of population j in region i (developing clinical immunity following infection)
- $\tau_i^j =$ number of treatments available to population j in region i. Note that this is a number as opposed to a rate.
- $\rho_i^j =$ average duration of immunity among population group j in region i;

Because the duration of immunity is extended through repeated exposure to parasite, the waning of immunity needs to account for the background entomological inoculation rate. Malaria modelling literature suggests that it should be modelled in the form [5-7]:

$$\omega_i^j = \frac{(\beta_i^j + \mu^j)\exp\left(-(\beta_i^j + \mu^j)\rho\right)}{1 - \exp\left(-(\beta_i^j + \mu^j)\rho_i^j\right)}$$

Model equations

The equations describing the human and mosquito models are as follows.

Human population

$$\frac{dS_i^j}{dt} = \left(\mu^j + \overline{\overline{\mu^j}}\right) * \left(pop_i^j\right) - \beta_i^j S_i^j + \min(\tau_i^j, I_i^j) + \omega_i^j R_i^j - \mu^j S_i^j$$

$$\frac{dE_i^j}{dt} = \beta_i^j S_i^j - \gamma E_i^j - \mu^j E_i^j$$

$$\frac{dI_i^j}{dt} = \gamma E_i^j - \min(\tau_i^j, I_i^j) - f_i^j I_i^j - (\mu^j + \Gamma^j) I_i^j$$

$$\frac{dR_i^j}{dt} = f_i^{\,j} I_i^j - \omega_i^j R_i^j - \mu^j R_i^j$$

Mosquito population

$$\frac{d\widehat{S_\iota}}{dt} = \hat{\mu} * (\widehat{pop_\iota}) - \widehat{\beta_\iota}\widehat{S_\iota} - \hat{\mu}\widehat{S_\iota}$$

$$\frac{dE_i}{dt} = \hat{\beta}_i \widehat{S_\iota} - \hat{\gamma}\hat{E}_i - \hat{\mu}\hat{E}_i$$

$$\frac{d\hat{I}_i}{dt} = \hat{\gamma}\hat{E}_i - \hat{\mu}\hat{I}_i$$

Programmatic responses considered and their implementation

The following programs and variations of programs were considered. Where possible, parameters were estimated for individual regions and population groups, to account for differences in the cost and effectiveness of delivering programs in different areas and to different people.

Reviews of published and grey literature were undertaken to obtain data for each program. Where no or limited estimates were available for specific regions or population groups, country estimates were used. Parameter estimates and references are shown in Table A1 and Table A2.

Long lasting insecticide treated nets (LLINs)

For each region and population group, LLIN programs have two features: coverage (the percentage that have an LLIN) and utilization (of those who have an LLIN, the percentage that use it). Studies have shown that in areas where they are used, LLINs can reduce the mosquito biting rate the mosquito biting rate N due to barrier protection; and the mortality rate among mosquitoes $\hat{\mu}$ due to insecticide treatment. If LLINs have effectiveness in reducing bites of ε_{LLIN}^{N} and they are treated with chemicals that have effectiveness ε_{LLIN}^{μ} at killing mosquitoes, then the biting rate for each population group and region is given by

$$\mathrm{N_i^j} = \underline{\mathrm{N}}_\mathrm{i}^\mathrm{j}\left(1 - \varepsilon_{\mathrm{LLIN}}^{\mathrm{N}}\right) * \mathrm{LLIN\ coverage_i^j} * \mathrm{LLIN\ utilization_i^j}$$

$$+\underline{\mathrm{N}}_\mathrm{i}^\mathrm{j}\left(1 - \mathrm{LLIN\ coverage_i^j} * \mathrm{LLIN\ utilization_i^j}\right)$$

and the mosquito mortality for each region is given by

$$\hat{\mu}_i = \underline{\hat{\mu}}\left(1 - \varepsilon^{\mu}_{LLIN}\right) * \frac{1}{\text{human pop}_i} \sum_j \text{LLIN coverage}^j_i * \text{LLIN utilization}^j_i * \text{pop}^j_i + \underline{\hat{\mu}}\left(1 - \frac{1}{\text{human pop}_i} \sum_j \text{LLIN coverage}^j_i * \text{LLIN utilization}^j_i * \text{pop}^j_i\right)$$

where the underlined $\underline{N}$ and $\underline{\hat{\mu}}$ values represent the biting rate and mosquito mortality rate in the absence of LLINs. Note that the biting rate applies to each population group whereas the mosquito mortality is regional.

Therefore the biting rates and mosquito mortality varies when the coverage or utilization of LLINs changes. The coverage of LLINs (in each region and among each population group) can be increased with additional spending on nets, and a logistic curve was used to relate spending to coverage which assumed a saturation value of 95% of the total population. The utilization of LLINs can be increased with increased coverage of a behavioural change and communication (BCC) program. This in turn requires additional spending on the BCC program; see section on the BCC program.

LLINs are required to be replaced every 5 years, and so we modelled the coverage to reduce by 1/5th each year.

Indoor residual spraying (IRS)

Coverage of IRS acts on two parameters in the model: the mosquito biting rate N, due to targeted killing of mosquitoes who approach humans; and the mortality rate among mosquitoes $\hat{\mu}$. If IRS has an effectiveness in reducing bites of ε^{N}_{IRS} and the chemicals used have an effectiveness ε^{μ}_{IRS} at killing mosquitoes, then the biting rate for each population group and region and mosquito mortality for each region are given by (similarly to LLINs):

$$N^j_i = \underline{N^j_i}\left(1 - \varepsilon^{N}_{IRS}\right) * \text{IRS coverage}^j_i + \underline{N^j_i}\left(1 - \text{IRS coverage}^j_i\right)$$

$$\hat{\mu}_i = \underline{\hat{\mu}}\left(1 - \varepsilon^{\mu}_{IRS}\right) * \frac{1}{\text{human pop}_i} \sum_j \text{IRS coverage}^j_i * \text{pop}^j_i + \underline{\hat{\mu}}\left(1 - \frac{1}{\text{human pop}_i} \sum_j \text{IRS coverage}^j_i * \text{pop}^j_i\right)$$

where the underlined $\underline{N}$ and $\underline{\hat{\mu}}$ values represent the biting rate and mosquito mortality rate in the absence of IRS. Note that in comparison to LLINs, we would expect $\varepsilon^{N}_{LLIN} > \varepsilon^{N}_{IRS}$, and $\varepsilon^{\mu}_{LLIN} < \varepsilon^{\mu}_{IRS}$.

The coverage of IRS (in each region and among each population group) can be increased with additional spending, and a logistic curve was used to relate spending to coverage which assumed a

saturation value of 95% of the total population. IRS needs to be renewed annually, and so every year the coverage reduces to zero unless ongoing spending occurs.

Chemoprophylaxis in pregnancy (IPTp)

Coverage of IPTp applies only to the population of pregnant women (i.e. j value corresponding to this population), and has both a coverage (the percentage of pregnant women who have one or more doses) and utilization (defined as the percentage of pregnant women who had at least three doses, among those who had at least one). It acts firstly to move a proportion $\varepsilon_{IPTp}^{clear}$ from the I and R compartments to the S compartment, and secondly to reduce the force of infection by a factor $(1 - \varepsilon_{IPTp}^{N})$ where ε_{IPTp}^{N} is the effectiveness of IPTp in reducing new infections among pregnant women. Therefore, the biting rate among pregnant women in each region is given by

$$\mathrm{N_i^{j=preg}} = \underline{\mathrm{N_i^{j=preg}}}\left(1 - \varepsilon_{IPTp}^{\mathrm{N}}\right) * \mathrm{IPTp\ coverage_i^{j=preg}} * \mathrm{IPTp\ utilization_i^{j=preg}} + \underline{\mathrm{N_i^{j=preg}}}\left(1 - \mathrm{IPTp\ coverage_i^{j=preg}} * \mathrm{IPTp\ utilization_i^{j=preg}}\right)$$

where the underlined $\underline{N}$ value represents the biting rate in the absence of IPTp. The coverage of IPTp (in each region) can be increased with additional spending, and a logistic curve was used to relate spending to coverage which assumed a saturation value of 95% of pregnant women. The utilization of IPTp can be increased with increased coverage of a BCC program. Increasing coverage of the BCC program in turn requires additional spending; see section on the BCC program. When utilized, IPTp is assumed to last for the duration of pregnancy.

Seasonal mass chemoprevention in children (SMC)

Coverage of SMC applies only to the population of children (i.e. j value corresponding to this population) and only applies to the North East and North West geopolitical zones, where malaria is highly seasonal. Similarly to IPTp, SMC acts firstly to shift a proportion $\varepsilon_{SMC}^{clear}$ of children from the I and R compartments to the S compartment, and secondly to reduce the force of infection by a factor $(1 - \varepsilon_{SMC}^{N})$ where ε_{SMC}^{N} is the effectiveness of SMC in reducing new infections among children. Therefore, the biting rate among children in each region is given by

$$\mathrm{N_i^{j=child}} = \underline{\mathrm{N_i^{j=child}}}\left(1 - \varepsilon_{SMC}^{\mathrm{N}}\right) * \mathrm{SMC\ coverage_i^{j=child}} + \underline{\mathrm{N_i^{j=child}}}\left(1 - \mathrm{SMC\ coverage_i^{j}}\right)$$

where the underlined $\underline{N}$ value represents the biting rate in the absence of SMC. The coverage of SMC (in each region) can be increased with additional spending, and a logistic curve was used to relate spending to coverage which assumed saturations values of 78% of children in the NW region, 50% of children in the NE region, 4% of children in the NC region and 0% of children in other regions, based on the proportion of each region that is suitable for the program (which requires seasonal malaria). When utilized, SMC is assumed to give protection for a year.

Mass drug administration (MDA)

Similarly to IPTp and SMC, acts firstly to shift a proportion $\varepsilon_{MDA}^{clear}$ of people from the I and R compartments to the S compartment, and secondly to reduce the force of infection by a factor $(1-\varepsilon_{MDA}^{N})$ where ε_{MDA}^{N} is the effectiveness of MDA in reducing new infections. Therefore, the biting rate among each population group and in each region is given by

$$\mathrm{N_i^j} = \underline{\mathrm{N_i^j}}(1-\varepsilon_{MDA}^{\mathrm{N}}) * \text{MDA coverage}_\mathrm{i}^\mathrm{j} + \underline{\mathrm{N_i^j}}\left(1-\text{MDA coverage}_\mathrm{i}^\mathrm{j}\right)$$

where the underlined $\underline{N}$ value represents the biting rate in the absence of MDA. The coverage of MDA (in each region) can be increased with additional spending, and a logistic curve was used to relate spending to coverage which assumed saturations values of 78% of the population in the NW region, 50% of the population in the NE region, 4% of the population in the NC region and 0% of the population in other regions, based on the proportion of each region that is suitable for the program.

Larval source management (LSM)

Coverage of LSM acts to reduce the population density of mosquitoes in the model. This in turn will reduce the biting rate of mosquitoes on the human population. If LSM has an effectiveness in reducing bites of ε_{LSM}^{N}, then the biting rate for each population group is given by:

$$\mathrm{N_i^j} = \underline{\mathrm{N_i^j}}(1-\varepsilon_{LSM}^{\mathrm{N}}) * \text{LSM coverage}_\mathrm{i}^\mathrm{j} + \underline{\mathrm{N_i^j}}\left(1-\text{LSM coverage}_\mathrm{i}^\mathrm{j}\right)$$

where the underlined $\underline{N}$ value represents the biting rate in the absence of LSM. The coverage of LSM (equal among all population groups) can be increased with additional spending, and a logistic curve was used to relate spending to coverage which assumed a saturation value of 25% of the total population given the need for areas to be suited.

Behavioural change and communication (BCC) program

Education campaigns in Nigeria are typically operated through churches and mosques or targeted to healthcare workers, and provide information on the proper use and importance of various malaria prevention and treatment programs. This includes correct usage and replacement of LLINs, in particular for high-risk groups such as children and pregnant women, as well as the benefits of programs like IPTp.

Changing the coverage of BCC programs influence several parameters in the model. For those who receive education, there has been a documented increase in utilization of LLINs of $\varepsilon_{BCC}^{LLIN\,ut} = 20\%$, and IPTp of $\varepsilon_{BCC}^{IPTp\,ut} = 30\%$ [8].

Coverage of BCC program was assumed to be nested. This means that as the coverage increased, it was assumed to do so among individuals who had access to LLINs or IPTp first, before reaching the population who were not covered by these programs. This was done in preference to random interactions, as it was believed that having access to prevention programs was likely to be correlated

with access to the education program. This also meant that if the coverage of the BCC program increased beyond the coverage of the LLIN or IPTp programs, then it had no additional effects on utilization in the model. A logistic curve was used to relate spending on BCC to coverage which assumed a saturation value of 95% of the total population.

Combining the effects of interventions

When multiple interventions were allocated to the same population they were assumed to do so at random; namely, the likelihood of an individual within a population receiving an intervention was independent of the coverage of other interventions. The exception to this was the BCC program, which was assumed to be preferentially provided to people who had LLINs or pregnant women who had started IPTp (Figure A2). Therefore, when multiple program coverages were changed, the change in mosquito biting rate, mosquito mortality were updated iteratively.

Model calibration and validation

For each population group and region, an optimization algorithm [9] was used to calibrate parameters for the biting rate (N_i^j), the proportion who develop immunity following infection $\left(f_i^j\right)$, the duration of immunity $\left(\omega_i^j\right)$, and the malaria specific mortality rate $\left(\Gamma_{\mathrm{i}}^j\right)$, so that at equilibrium the model outcomes best fit available data for the 2015 malaria incidence, mortality and *Plasmodium falciparum* prevalence. The algorithm was based on minimizing the sum of squared errors between the modelled outcomes and the data.

The short malaria lifecycle and rapid changes to intervention coverage over the previous five years dominates any background trends in incidence and mortality, making it suitable to start a model from equilibrium for forward projections. However, the effects of changes in intervention coverage on epidemiological outcomes still needed to be validated. This was done by separately calibrating the model to 2010 data and program coverages (the only other year data was available), and then linearly changing the coverage of programs to 2015 values while running the model over this five-year period. The resulting model estimates for 2015 incidence, prevalence and malaria-attributable deaths among each region and population group were then compared to 2015 data estimates. Model calibration results are shown in Figure A2.

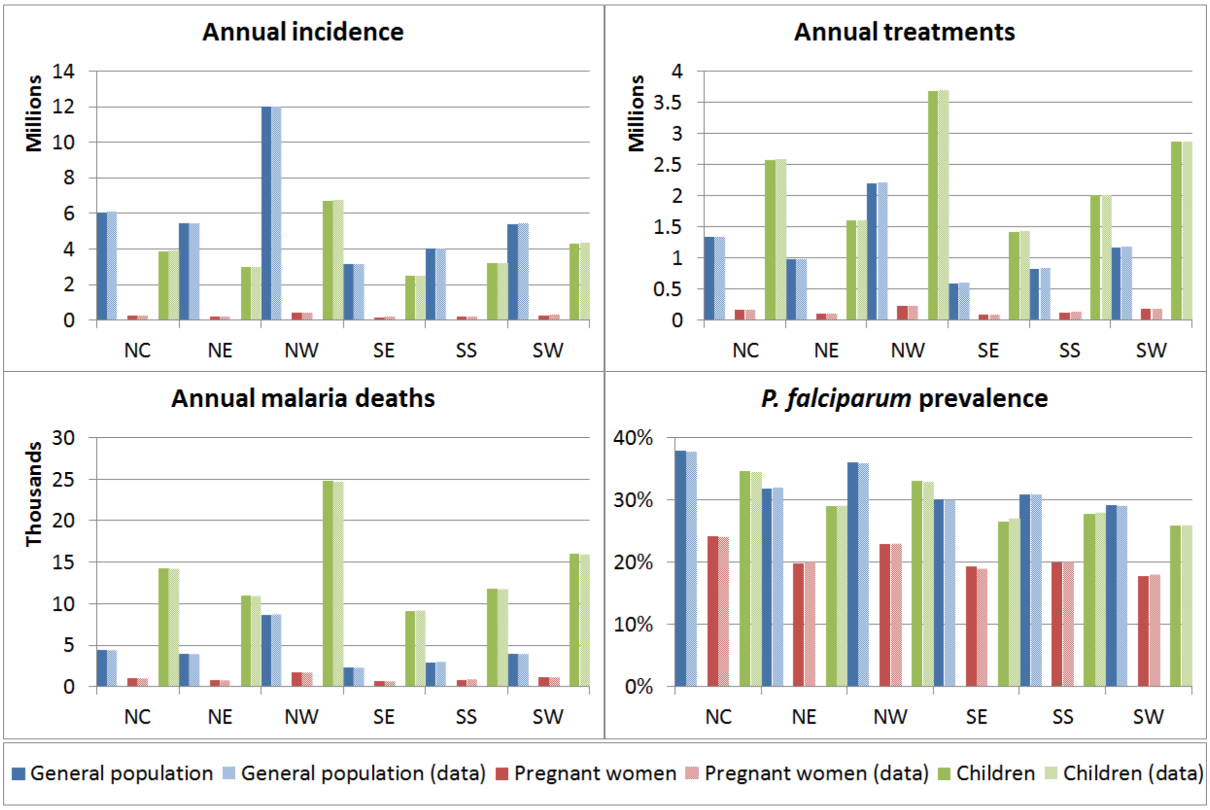

Figure A2: Model calibration. 2015 data versus modelled incidence (top left), treatment numbers (top-right), malaria-attributable mortality (bottom-left) and *P. falciparum* prevalence (bottom-right) for each population group and region.

Cost-coverage curves

The key assumptions of resource optimization are the relationships between (a) the cost of prevention and treatment programs; (b) the resulting coverage levels among targeted populations; and (c) how these coverage levels influence clinical and epidemiological outcomes. The relationship between (b) and (c) is described above for each program, however the link between (a) and (b) is also required to understand how incremental changes in spending are likely to affect outcomes (e.g. total incidence and total mortality).

Most programs typically have a period of effective scale-up as funding is increased, but attaining very high coverage levels involves reaching the most difficult to reach groups, which requires increased incremental investment for demand generation and related activities (i.e. there is a saturation effect with increased funding). These effects are incorporated in the model using logistic function cost-coverage curves (Figure A3). Logistic functions allow coverage to saturate at high spending levels, thus better reflecting the program reality. The relationship between spending and coverage was therefore calculated as

$$C(x) = \left(\frac{2B}{1 + \exp\left(-\frac{2x}{PBu}\right)} - B \right) P$$

where C(x) is the number of people covered for a given amount of budget x; B is the saturation coverage (upper asymptote of Figure A3), P is the target population size and u is the unit cost. Changes in coverage expected from changes in program funding are assumed by interpolating and extrapolating available data using the fitted logistic curve. These are in turn related to changes in the model parameters as described in the programmatic responses section above.

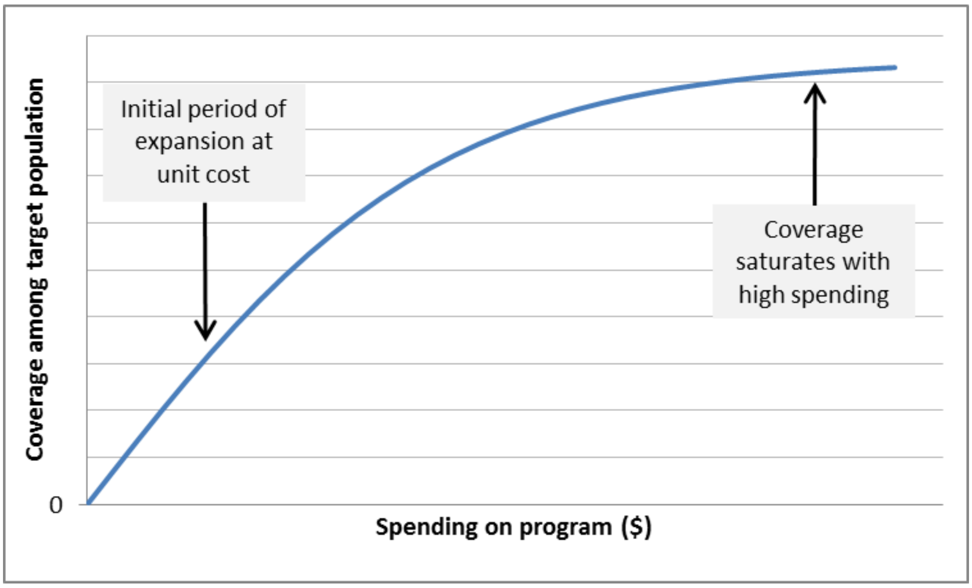

Figure A3: Example logistic cost-coverage curve. When spending on a program becomes very high, only marginal gains in coverage can be made as a saturation effect occurs.

Optimization

Regional specific optimizations

For each region, an existing optimization algorithm [9] was used to incrementally shift the current available budget (Table C1) between programs (treatment, LLINs, IRS, IPTp, SMC, MDA, LSM) and population groups (children 0-5 years, pregnant women, general population) in order to achieve (a) a minimum 5-year incidence, and (b) a minimum 5-year mortality. This was then repeated for different total available budgets.

Geographical optimization

Once the regional estimates had been completed, a cost-effectiveness curve could be formed for each region. These curves describe the additional incidence (or mortality) averted for incremental increases in total funding to each region (Figures D8-D9). The same optimization algorithm was then used to minimize (a) total country-level incidence and (b) total country-level mortality by shifting funding between regions according to the relative cost-effectiveness ratios for each region. Where total funding within a region changed, it was allocated to programs and population groups optimally according to the region specific optimizations.

Sensitivity analysis

Once the geospatially optimal allocation of funding was determined for each scenario, a multivariate sensitivity analysis was conducted to estimate bounds for the impact of allocating funding in this way (i.e. bounds for the number of deaths and cases prevented). 95% bounds were obtained for the number of deaths or cases averted using Monte Carlo sampling for the model's structural parameters ($\mu, \hat{\mu}, \gamma, \hat{\gamma}$), unit costs and the effects of programs, with samples taken from Normal distributions around point estimates (standard deviations of 5%, truncated at 10% above and below).

Appendix B: Model parameters

Table B1: Model parameters, values and sources

Symbol	Parameter	Estimated for (where data available; default assumption is no variation between regions and populations)	Value	Notes
HUMANS				
	Population size	Each region and population group	Table B3	
μ_i^j	All-cause mortality rates	Each region and population group	Table B3	
Γ_i^j	Additional mortality rate due to malaria	Each region and population group	—	Calibration parameter
N_i^j	Mosquito biting rate	Each region and population group	—	Calibration parameter
λ	Probability of transmission from (infected) mosquito to human per bite	Global parameter	5%	[10]
γ	1/average duration of latency	Global parameter	1/9.9 days	[11, 12]
f_i^j	Recovery rate for developing clinical immunity	Each region and population group	—	Calibration parameter
ρ_i^j	Average duration of immunity	Each region and population group	—	Calibration parameter
τ_i^j	Treatment numbers	Each region and population group	Table B3	
MOSQUITOES				
	Population size	Each region		
$\hat{\mu}$	1/life expectancy	Global parameter	1/5 weeks	[13, 14]
$\widehat{N_\iota}$	Biting rate	Each region and population group, equal to human rate	—	As above and in transmission section, calibration parameter
$\hat{\lambda}$	Probability of transmission from (infected) human to mosquito per bite	Global parameter	47%	[7, 12, 15-17]
γ	1/average duration of latency	Global parameter	1/14 days	[14, 18]
INTERVENTIONS				
ε_{LLIN}^N	Effectiveness of LLIN in reducing bites	Global parameter	56%	[19], and during pregnancy [20]. Note Cochrane review has LLIN reducing incidence by 50% [21], not

				necessary bites.
ε_{LLIN}^{μ}	Effectiveness of LLIN in killing mosquitoes	Global parameter	19%	Treatments kill 41% of the 46% of mosquitoes not repelled that landing on net [19, 22]. Note that this is independent of the reduction in mosquito bites.
ε_{IRS}^{N}	Effectiveness of IRS in reducing bites	Global parameter	30%	[19]. Note Cochrane review has LLIN reducing incidence by 50% [23], not necessary bites.
ε_{IRS}^{μ}	Effectiveness of IRS in killing mosquitoes	Global parameter	56%	IRS kills 80% of the 70% of mosquitoes not repelled [19, 22]
$\varepsilon_{IPTp}^{clear}$	Proportion of pregnant women carrying parasites who clear them	Global parameter	95%	[19, 24]
ε_{IPTp}^{N}	Effectiveness of IPTp in preventing new infections in pregnant women	Global parameter	90%	[19]
$\varepsilon_{SMC}^{clear}$	Proportion carrying parasites who clear them	Global parameter	95%	[19, 24]
ε_{SMC}^{N}	Effectiveness of SMC in preventing new infections in children	Global parameter	50%	Malaria Control State Fact Sheet for state of Katsina [25]. Also 47% effectiveness estimated in 2008 Cochrane review [26]. Efficacy in randomized controlled trials ranges from 75-87% [27, 28].
$\varepsilon_{MDA}^{clear}$	Proportion carrying parasites who clear them	Global parameter	95%	[19]
ε_{MDA}^{N}	Effectiveness of MDA in preventing new infections	Global parameter	90%	[19]
ε_{LSM}^{N}	Effectiveness of LSM in reducing the biting rate	Global parameter	52%	[29]
$\varepsilon_{BCC}^{LLIN\ ut}$	Effectiveness of BCC in increasing LLIN utilization	Global parameter	20%	Effects would be highly variable based on local context and implementation, with these values cautiously estimated from the higher

				reported success of one faith-based program[8]
$\varepsilon_{BCC}^{IPTp\ ut}$	Effectiveness of BCC in increasing IPTp utilization	Global parameter	30%	Effects would be highly variable based on local context and implementation, with these values cautiously estimated from the higher reported success of one faith-based program[8]

Table B2: Estimated unit costs of programs

Program	Unit cost (cost per person)	Source	Comment
Treatment	Child course = $0.65; Adult course = $1.14	[30, 31]	Department for International Development (DFID) artemisinin combination therapy procurement costs (procured through the Global Fund's Affordable Medicines Facility for malaria (AMFm) programme). Price varies depending on age category.
Diagnostic tests	$1.95	[31]	Health sector cost per service. Weighted cost from 2014 ratio of 15% of tests by microscopy = $1.87 and 85% by rapid diagnostic test = $1.97.
LLIN	$2.61	[32] [30] [33]	One net for two people is defined as full coverage (Nigeria Malaria Indicator Survey [32]); $4.71 is the cost per LLIN for 2016 determined by the Global Fund ($2.55 per net and $2.16 for distribution, consistent with the DFID [30]) divided by 1.8 as recommended by the WHO in terms of procurement because of odd numbered person households to ensure enough for a mass distribution [33].<br>$4.71 / 1.8 = $2.61 per person covered.
IRS	$2.38	[34]	Unit cost per household for the four year period between 2013 and 2016 calculated at NGN1,884 (IRS Quantification and Budget 2013-2016 Final [34]); 2014 exchange rate 1USD = 158.55NGN [35]; divided by 5 for the standard household size.
IPTp	$1.10	[36]	President's Malaria Initiative 2015 reports $2.2 million spent on IPTp for 4 million doses of sulfadoxine-pyrimethamine suggesting a cost of $1.10 per person. This is consistent with White et al. [37].
SMC	$1.75	[37]	Median cost of a years worth of protection was $4.03; however only 13% of this cost was for the treatment (the rest being for infrastructure and personnel) [37].
LSM	$1.65	[38]	Cost of a years' worth of maintenance in suitable areas. Unit cost for larviciding includes some other potentially cheaper larval control methods like reengineering of irrigation to have less open standing water.
BCC	$0.03	[8]	NIFAA program trained 6,500 faith leaders; when the total NIFAA budget is divided by 2/3 (for the 2 out of 3 states where implementation took place), the cost to reach and train each faith leader was estimated to be $0.65. Assuming 20 contacts per faith leader, then the cost per exposure would be $0.65/20 = $0.033.
MDA	$5.25	[19]	Walker et al. [19] found the cost in 2012 of three rounds of seasonal malaria chemoprevention using door-to-door delivery was estimated at $6.10 in Senegal and $4.40 in Mali; we used the average of these two estimates.

Data estimates for each region and population group

The data used to populate the model (population size, population growth rates and all-cause mortality rates) and calibrate the model (incidence, prevalence, testing, treatment and malaria-attributable deaths) is shown for each region and population group in Table B3. This data was estimated based on information from a range of sources, as described below.

The total population of each state (and hence geopolitical region) in 2015 was obtained from the Malaria Atlas Project [39, 40]. This was then divided among population groups: the number of children 0-4 years (inclusive) as a percentage of the total Nigerian population was obtained from UN Population Data [1, 2] from 2000 to 2014, and used to estimate the number of children in each region. This was between 17.2% and 17.5% of the total population depending on the year. The number of pregnant women was estimated using the crude birth rate for Nigeria [41]. The general population size was then taken as the remaining population. UN Population Data [1, 2] from 2000 to 2014 was used to estimate a linear annual population growth rate.

Incidence data (for calibration purposes) was estimated using different methods for each population group. For incidence among children, methods from Cameron et al. [42] were used, which establishes a relationship between the infection prevalence of *P. falciparum* malaria in Children 2-10 (as reported in the Malaria Atlas Project) and incidence in Children 0-5. This worked out to a linear conversion where for example 60% prevalence converted to 1.5 cases of malaria per child per year. For pregnant women, this was based on the assumption that pregnant women have the same incidence to prevalence ratio as children from Cameron et al. [42], with the prevalence among pregnant women determined by the ratio of 1/1.44 for prevalence in children 0-5 to prevalence in pregnant women implied in van Eijk et al. [43]. Finally, the general population incidence was taken as the total incidence for each region from the Malaria Atlas Project [39, 40] minus the incidence among children and pregnant women.

Prevalence among children 0-5 years was assumed to be equal to 2015 *P. falciparum* prevalence among children 2-10 years from the Malaria Atlas Project [39, 40]. Prevalence among pregnant women was then calculated by the ratio of 1/1.44 for prevalence in children 0-5 to prevalence in pregnant women implied in van Eijk et al. [43]. No estimates were available for prevalence among the general population, and in particular, Cameron et al. found no correlation between incidence and prevalence [42]. Therefore, prevalence in the general population was estimated as: (proportion of national child cases in this region * (number of estimated national cases not accounted for by child and pregnant women estimates) / region population.

Number of tests was estimated as incidence multiplied by the percentage of children 0-5 years with a fever who are tested in each state [44] and a weighting factor for each population (we assume that adults with a fever are one third as likely to be tested as children and pregnant women), then normalised across regions to the known total number of tests for all of Nigeria in 2015 from the World Malaria Report [44].

Treatment numbers were estimated as incidence multiplied by the percentage of children 0-5 with a fever who are given (any) malaria treatment in each region [44] and a weighting factor for each population (we assume that adults with a fever are a third less likely to be tested than children and

pregnant women), then normalised across regions to the known total number of treatments for Nigeria in 2015 from the World Malaria Report [44].

All-cause mortality rates were taken from UN Population Division data [2]. Regional adult 15-60 mortality rates were scaled for general population 6+ to match national all-cause mortality rate after removing estimated maternal and child mortality. Maternal mortality added for pregnant women.

Malaria-attributable deaths were based on the WHO World Malaria Report 2015 [44] estimates for Nigeria in 2014 (119,000) and their estimate that 73% (292,000 out of 400,000) of deaths in the African Region are in children. These estimated 86,870 child 0-5 deaths were distributed across regions according to the relative *P. falciparum* prevalence values reported in the Malaria Atlas Project [39, 40] (not taking into account that some states are likely to have better treatment and lower mortality for the same number of cases, as no data were available). The remaining deaths were distributed among the general population and pregnant women populations in each region according to their population sizes and relative all-cause mortality rates (which included malaria-attributable deaths).

Table B3: Regional data estimates for 2015 used for the modelling

	NC	NE	NW	SE	SS	SW	***Total / weighted average***
Children 0-5 years							
Population size	4,475,710	4,116,285	8,126,256	3,651,727	4,531,168	6,629,081	*31,530,227*
Annual population growth rate [1, 2]	2.56%	2.46%	2.69%	2.80%	2.51%	3.56%	*2.81%*
Incidence	3,872,538	2,984,305	6,732,488	2,484,119	3,199,273	4,354,922	*23,627,645*
Prevalence	35%	29%	33%	27%	28%	26%	*30%*
Testing	1,292,330	721,398	1,368,209	584,546	745,987	2,710,982	*7,423,452*
Treatment	2,573,541	1,603,655	3,680,725	1,423,092	2,004,220	2,867,914	*14,153,147*
All-cause mortality rates (including malaria-attributable deaths)	2.00%	3.20%	3.70%	2.62%	1.82%	1.80%	*2.60%*
Malaria-attributable deaths	14,238	10,972	24,753	9,133	11,763	16,011	*86,870*
General							
Population size	21,179,177	19,478,369	38,453,653	17,280,069	21,441,603	31,368,985	*149,201,856*
Annual population growth rate [1, 2]	2.56%	2.46%	2.69%	2.80%	2.51%	3.56%	*2.81%*
Incidence	6,056,436	5,430,025	11,976,341	3,151,662	4,017,444	5,443,833	*36,075,741*
Prevalence	38%	32%	36%	30%	31%	29%	*33%*
Testing	673,711	437,534	811,297	247,209	312,254	1,129,613	*3,611,618*
Treatment	1,334,480	976,423	2,201,110	596,752	825,806	1,172,247	*7,106,818*
All-cause mortality rates (including malaria deaths)	1.08%	1.06%	1.13%	0.96%	0.91%	0.79%	*0.99%*
Malaria-attributable deaths	4,389	3,935	8,680	2,284	2,912	3,945	*26,145*
Pregnant women							

Population size	403,430	371,032	732,481	329,158	408,429	597,530	*2,842,060*
Annual population growth rate [1, 2]	2.56%	2.46%	2.69%	2.80%	2.51%	3.56%	*2.81%*
Incidence	242,404	186,804	421,424	155,495	200,260	272,599	*1,478,986*
Prevalence	24%	20%	23%	19%	20%	18%	*21%*
Testing	80,894	45,156	85,644	36,590	46,695	169,695	*464,674*
Treatment	161,092	100,382	230,397	89,079	125,455	179,519	*885,924*
All-cause mortality rates (including malaria-attributable deaths)	3.50%	3.48%	3.55%	1.58%	1.52%	1.40%	*2.56%*
Malaria-attributable deaths	981	756	1,705	629	810	1,103	*5,984*

Appendix C: Estimated current spending

Based on the unit costs of each malaria prevention or treatment program (**Error! Reference source not found.**), the estimated annual direct cost associated with the current coverage of programs (estimated via the logistic curves, Figure A3) was US$175,351,471 (Table C1). By comparison, the World Malaria Report records that in 2014 the Nigerian government reported direct malaria funding totalling US$285,931,583 (entirely donor-funded: Global Fund US$137,920,815; the World Bank US$52,220,588; PMI/USAID US$73,771,000; other bilaterals US$20,157,565; WHO US$861,615; and UNICEF US$1,000,000) [44]. There are several key factors that may explain the difference between our estimates and the 2014 value from the World Malaria Report. Firstly, out estimate does not include the costs of non-direct programs such as central management and surveillance. Second, for LLINs we have assumed that the cost of achieving this level of coverage was spread evenly over the past five years (given their five-year lifespan). If the majority of currently owned LLINs were purchased in more recent years, then the expenditure in these years would be considerably higher. Third, given the funding for malaria in Nigeria is entirely from donors, there is likely to be substantial variability between years.

The program receiving the greatest amount of funding was LLINs, followed by treatment, while the North West region was the one receiving the most current funding for programs.

Table C1: Estimated current annual spending by region, population group and program, according to coverage. Sources: total region population sizes (added to give regional values) from the Malaria Atlas Project [39, 45]; the percentage of these regional populations who are children from United Nations, Department of Economic and Social Affairs, Population Division (17%) [1]; and the percentage of these populations who are pregnant women (1.6%) based on Nigerian birth rate [41].

	Unit Cost	NW	NC	NE	SW	SE	SS
**General population**							
Population size		21,179,177	19,478,369	38,453,653	17,280,069	21,441,603	31,368,985
Diagnostic tests per year[a] + treatments per year[b]	$1.95+$1.14	$4,209,793	$3,078,591	$ 6,941,904	$1,880,599	$2,602,717	$3,694,485
LLIN (people covered)[c]	$0.52	$5,181,147	$8,662,494	$25,545,278	$11,951,832	$6,567,161	$5,508,221
IRS (people covered)[d]	$2.38	$496,162	$1,940,132	$ 5,748,304	$3,497,633	$3,016,357	$826,742
BCC (people covered)	$0.03	$223,279	$254,191	$563,129	$311,803	$345,181	$603,995
Estimated total annual spending		*$10,110,381*	*$13,935,409*	*$38,798,615*	*$17,641,867*	*$12,531,416*	*$10,633,443*
**Pregnant women**							
Population size		403,430	371,032	732,481	329,158	408,429	597,530
Diagnostic tests per year[a] + treatments per year[b]	$1.95 + $1.14	$540,956	$325,192	$754,165	$288,583	$409,890	$585,555
LLIN (people	$0.52	$153,114	$308,786	$992,718	$128,199	$179,047	$241,655

covered)[c]							
IPTp (people covered)	$1.10	$50,276	$65,988	$ 98,554	$59,126	$66,989	$148,491
BCC (people covered)	$0.03	$ 4,253	$ 4,842	$ 10,727	$ 5,939	$ 6,575	$11,505
Estimated total annual spending		*$748,599*	*$704,808*	*$ 1,856,163*	*$481,846*	*$662,501*	*$987,205*
**Children**							
Population size		4,475,710	4,116,285	8,126,256	3,651,727	4,531,168	6,629,081
Diagnostic tests per year[a] + treatments per year[b]	$1.95+ $0.65	$7,783,966	$4,447,126	$10,453,537	$3,946,559	$5,667,352	$8,078,479
LLIN (people covered)[c]	$0.52	$1,875,366	$2,699,278	$10,368,516	$1,822,227	$2,146,226	$2,284,037
SMC (people covered)	$1.75	$ 0	$ 0	$ 4,200,165	$ 0	$ 0	$ 0
BCC (people covered)	$0.03	$47,185	$53,717	$119,004	$65,892	$72,946	$127,640
Estimated total annual spending		*$9,706,517*	*$7,200,122*	*$25,141,221*	*$5,834,678*	*$7,886,523*	*$10,490,156*
**Combined populations**							
Estimated total annual spending		*$20,565,498*	*$21,840,339*	*$65,796,000*	*$23,958,391*	*$21,080,439*	*$22,110,805*
Country level estimated total[d]		*$175,351,471*					

[a] Total country level diagnostic tests were available (1,973,317) [44], assumed to be distributed in the same proportion as treatments across regions and population groups.

[b] Treatment numbers were calculated as per Appendix B.

[c] Assumes that LLINs last 5 years, and so current annual spending is estimated at 1/5th the cost per person covered.

[d] Includes coverage of pregnant women and children as well, assuming they are all covered proportionately.

[d] Does not include fixed costs such as central management and surveillance.

Appendix D: Additional charts

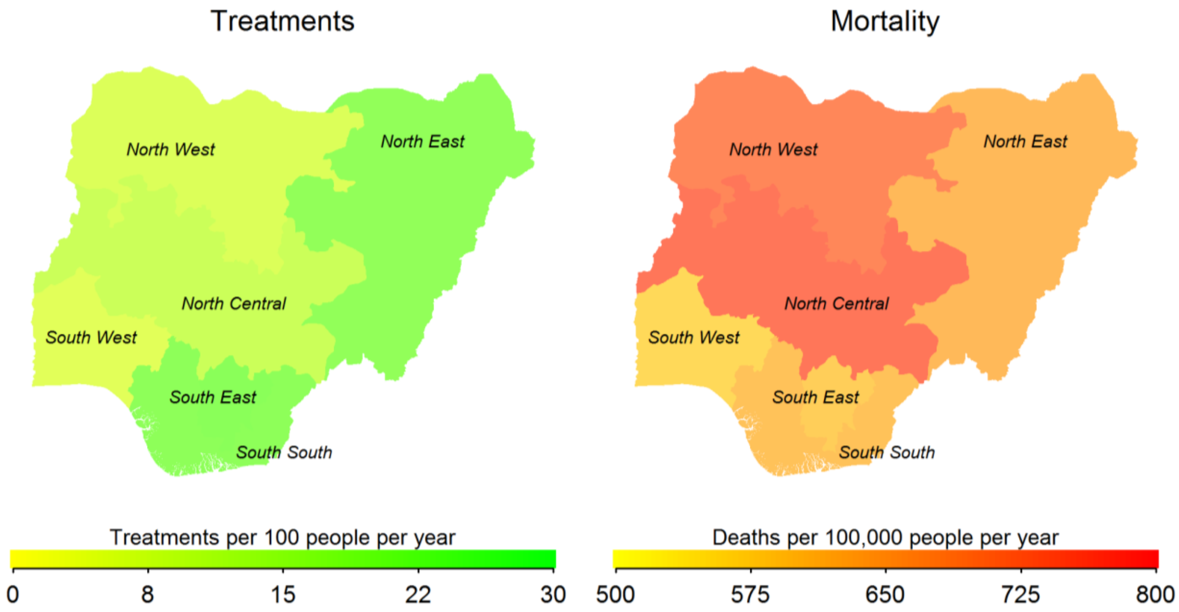

Figure D1: Annual population-weighted mortality and treatment numbers for malaria in Nigeria.

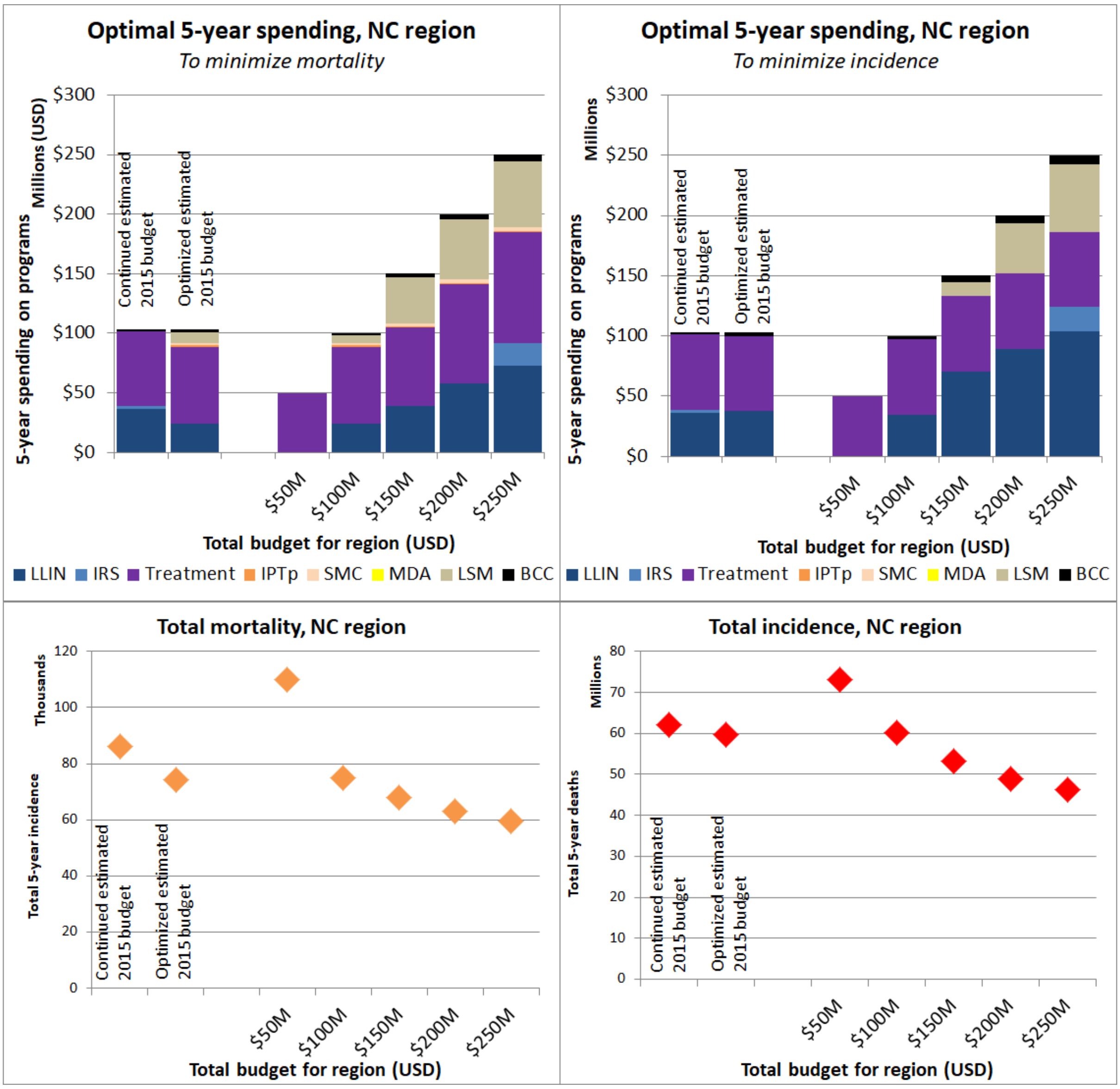

Figure D2: Estimated current and optimal 5-year spending allocations on programs in the North Central (NC) region for varying budget levels. Optimized to minimize malaria-attributable mortality (left) or incidence (right). Abbreviations: BCC, behavioural change communication; IPTp, intermittent presumptive treatment during pregnancy; IRS, indoor residual spraying; LLINs, long lasting insecticide treated nets; LSM, larval source management; MDA, mass drug administration; SMC, seasonal mass chemoprevention in children.

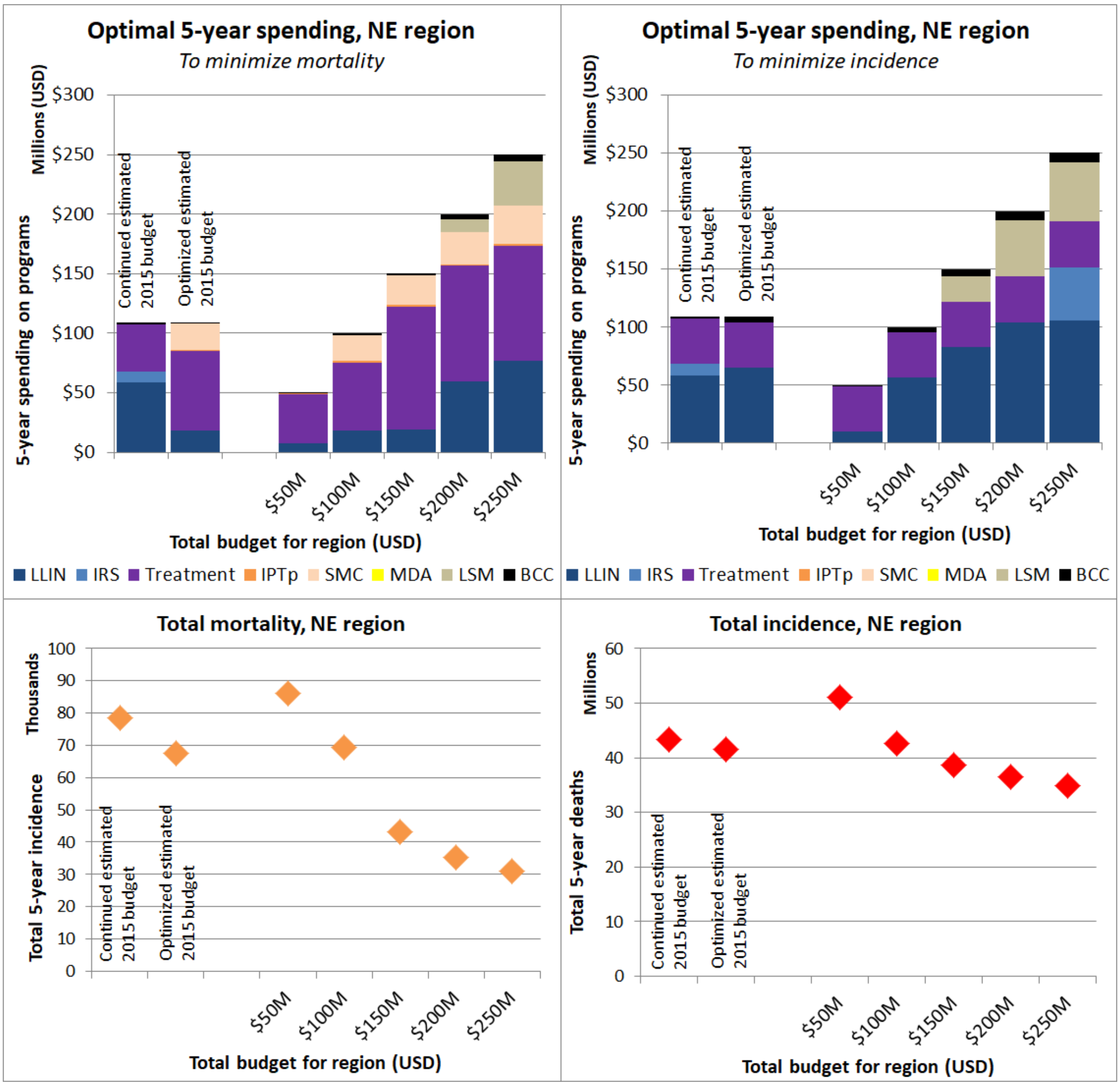

Figure D3: Estimated current and optimal 5-year spending allocations on programs in the North East (NE) region for varying budget levels. Optimized to minimize malaria-attributable mortality (left) or incidence (right). Abbreviations: BCC, behavioural change communication; IPTp, intermittent presumptive treatment during pregnancy; IRS, indoor residual spraying; LLINs, long lasting insecticide treated nets; LSM, larval source management; MDA, mass drug administration; SMC, seasonal mass chemoprevention in children.

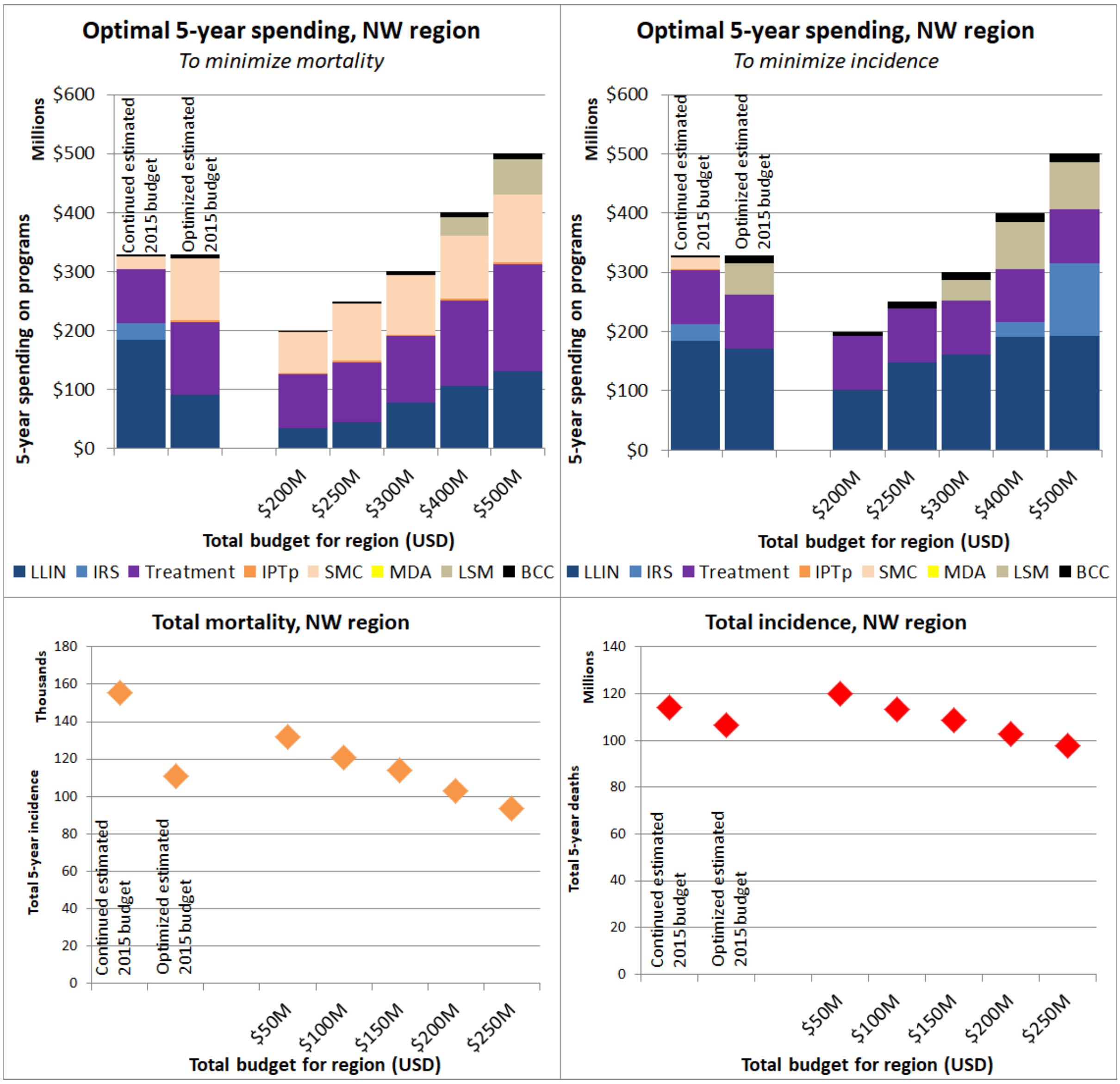

Figure D4: Estimated current and optimal 5-year spending allocations on programs in the North West (NW) region for varying budget levels. Optimized to minimize malaria-attributable mortality (left) or incidence (right). Abbreviations: BCC, behavioural change communication; IPTp, intermittent presumptive treatment during pregnancy; IRS, indoor residual spraying; LLINs, long lasting insecticide treated nets; LSM, larval source management; MDA, mass drug administration; SMC, seasonal mass chemoprevention in children.

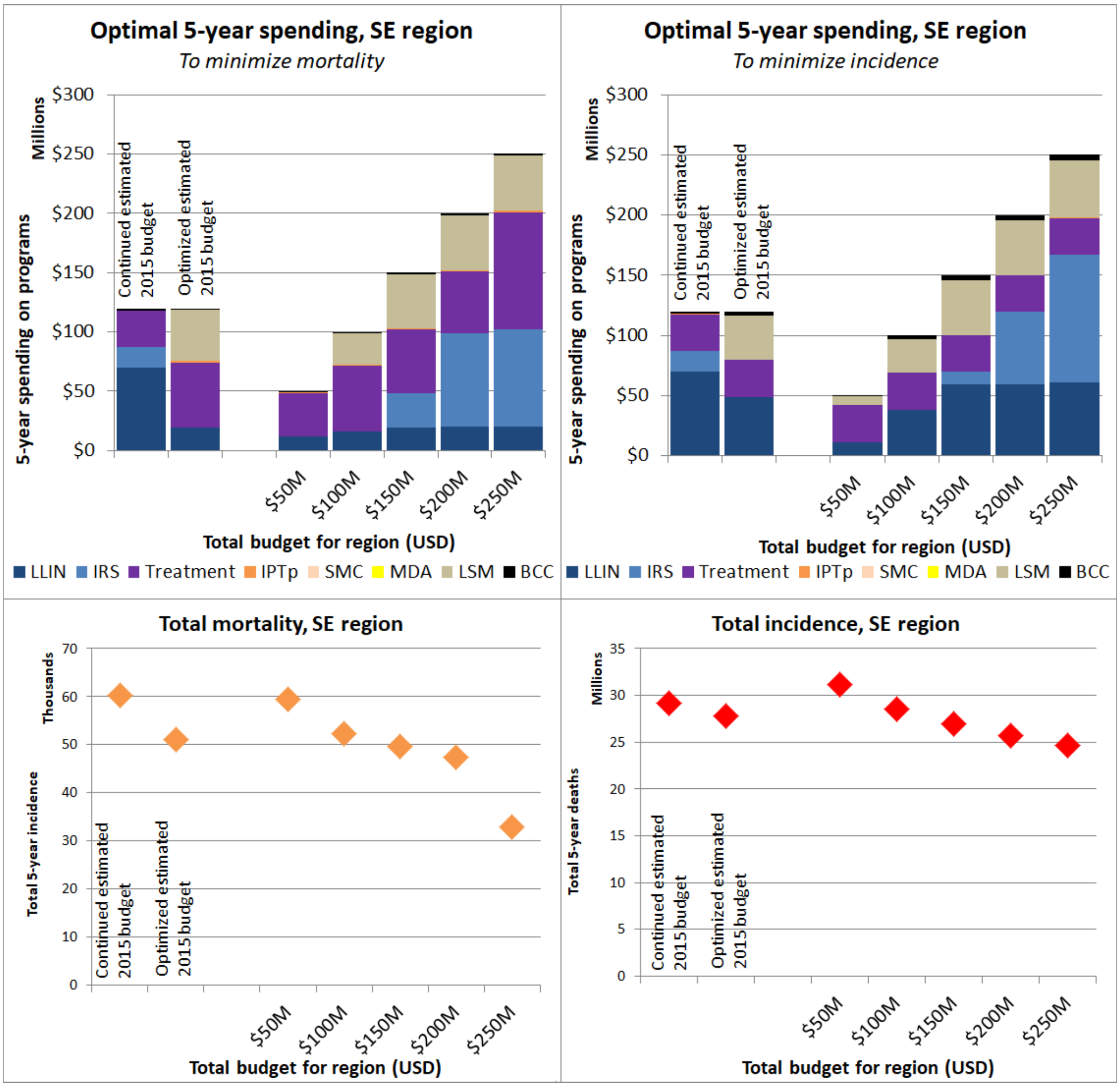

Figure D5: Estimated current and optimal 5-year spending allocations on programs in the South East (SE) region for varying budget levels. Optimized to minimize malaria-attributable mortality (left) or incidence (right). Abbreviations: BCC, behavioural change communication; IPTp, intermittent presumptive treatment during pregnancy; IRS, indoor residual spraying; LLINs, long lasting insecticide treated nets; LSM, larval source management; MDA, mass drug administration; SMC, seasonal mass chemoprevention in children.

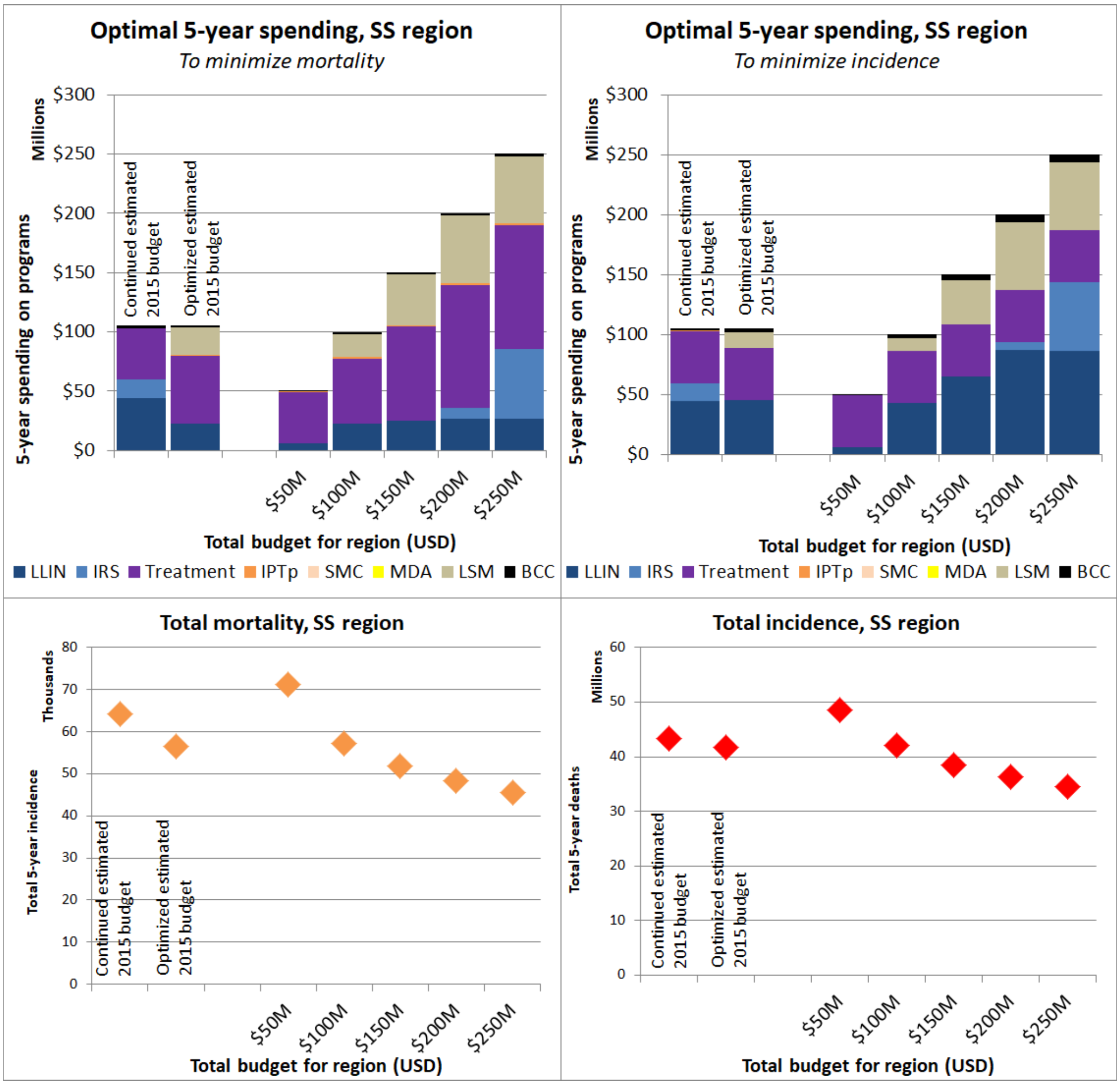

Figure D6: Estimated current and optimal 5-year spending allocations on programs in the South South (SS) region for varying budget levels. Optimized to minimize malaria-attributable mortality (left) or incidence (right). Abbreviations: BCC, behavioural change communication; IPTp, intermittent presumptive treatment during pregnancy; IRS, indoor residual spraying; LLINs, long lasting insecticide treated nets; LSM, larval source management; MDA, mass drug administration; SMC, seasonal mass chemoprevention in children.

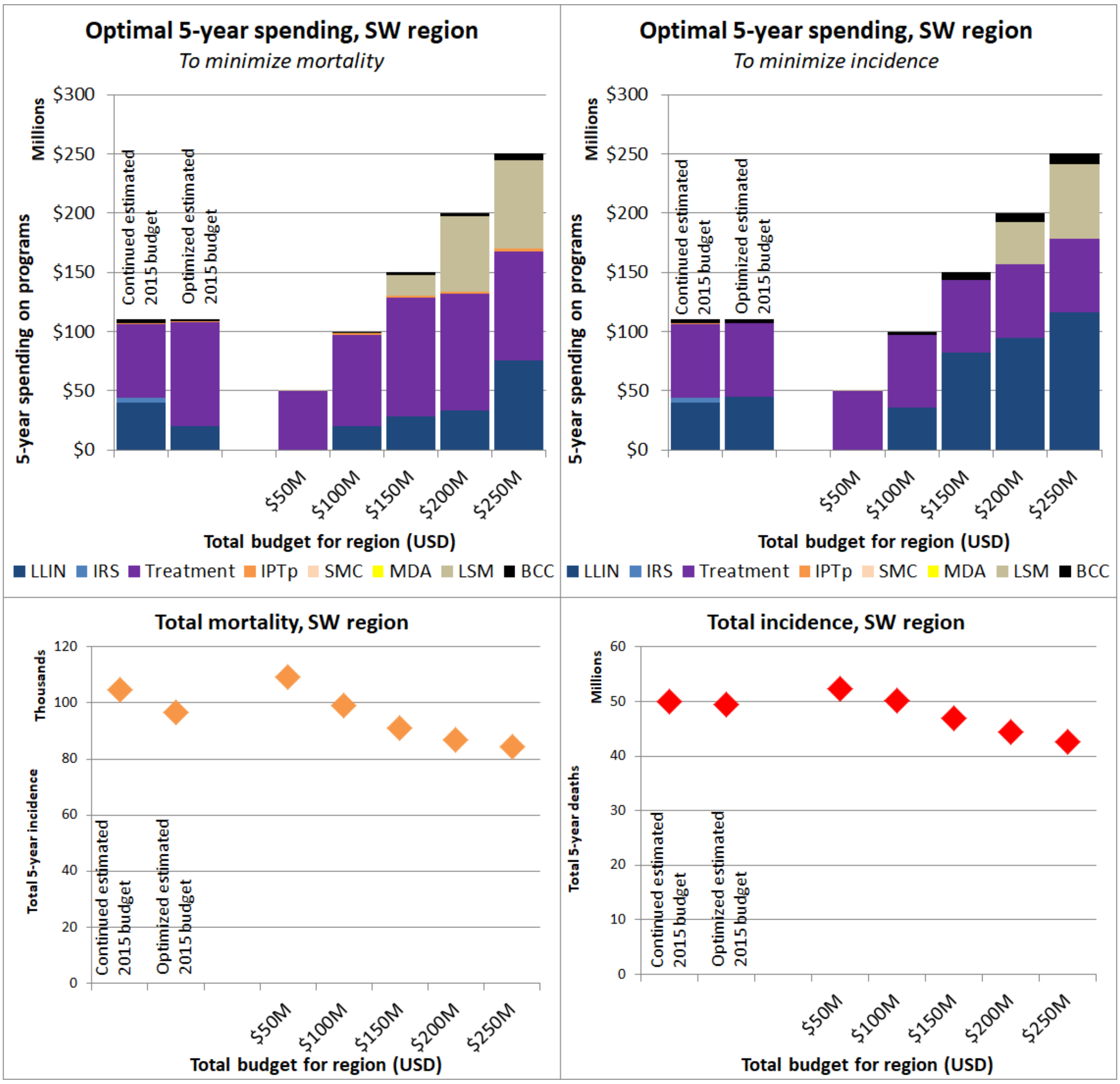

Figure D7: Estimated current and optimal 5-year spending allocations on programs in the South West (SW) region for varying budget levels. Optimized to minimize malaria-attributable mortality (left) or incidence (right). Abbreviations: BCC, behavioural change communication; IPTp, intermittent presumptive treatment during pregnancy; IRS, indoor residual spraying; LLINs, long lasting insecticide treated nets; LSM, larval source management; MDA, mass drug administration; SMC, seasonal mass chemoprevention in children.

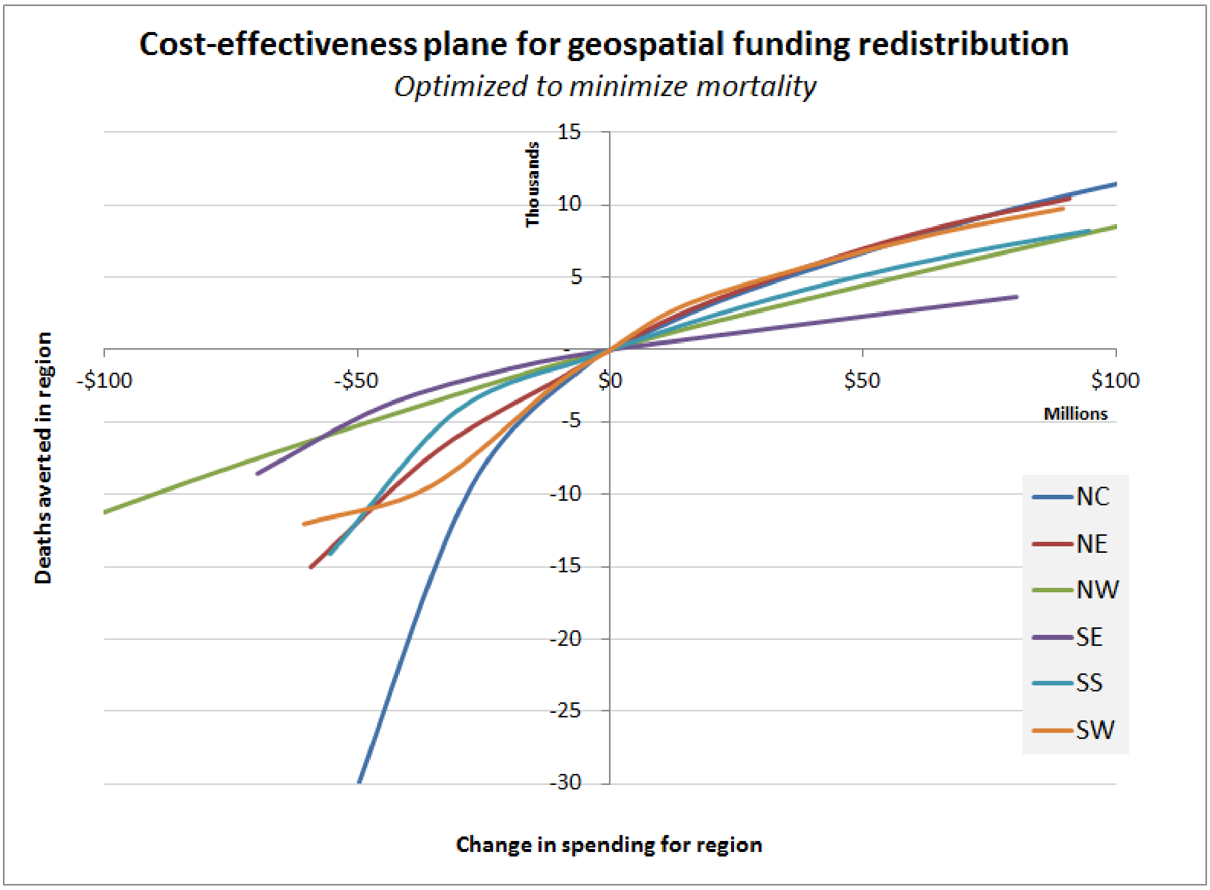

Figure D8: Cost-effectiveness plane to determine the optimal redistribution of funding between regions. Optimizations minimizing mortality. Curves for each region are based on the relationships between additional funding and total deaths shown in Figures D2-D7 (left).

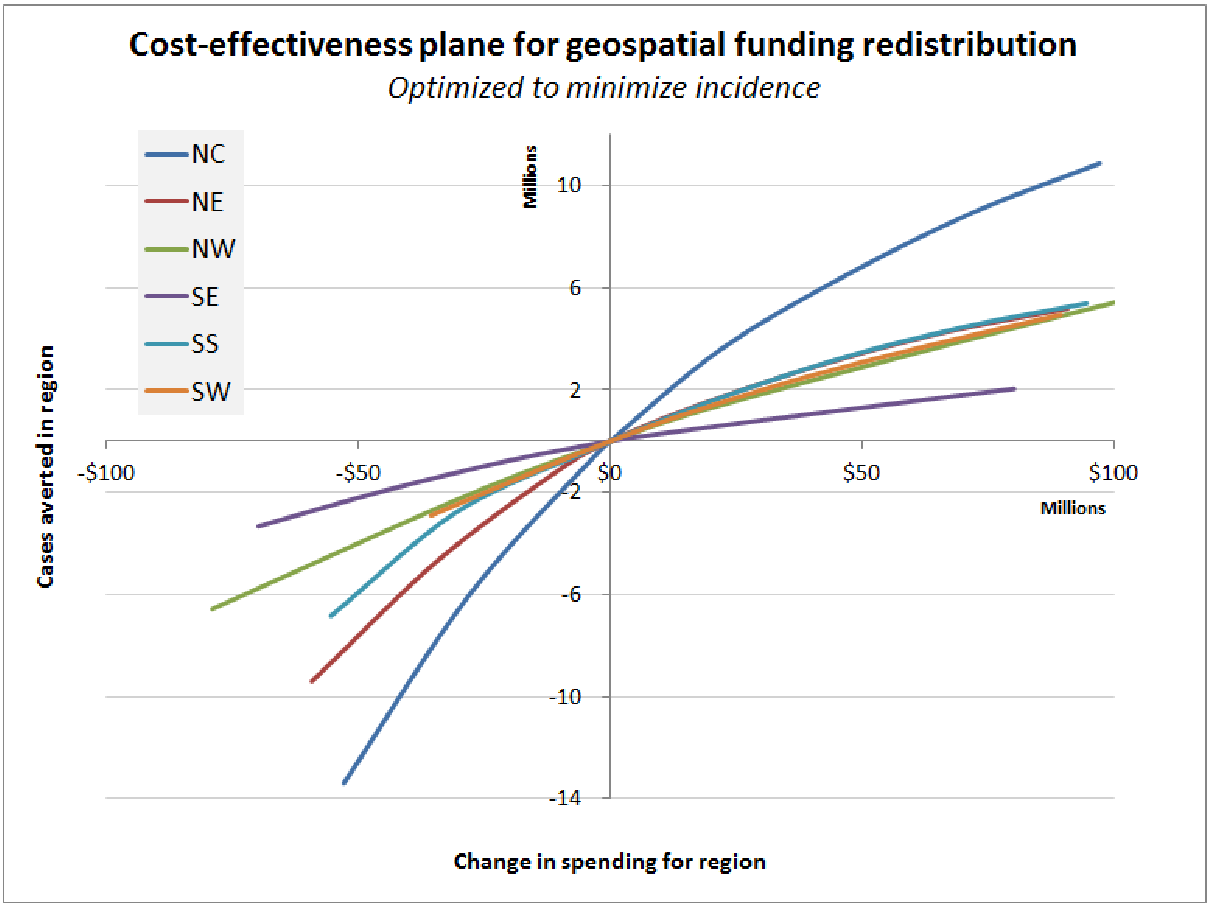

Figure D9: Cost-effectiveness plane to determine the optimal redistribution of funding between regions. Optimizations minimizing incidence. Curves for each region are based on the relationships between additional funding and total incidence shown in Figures D2-D7 (right).

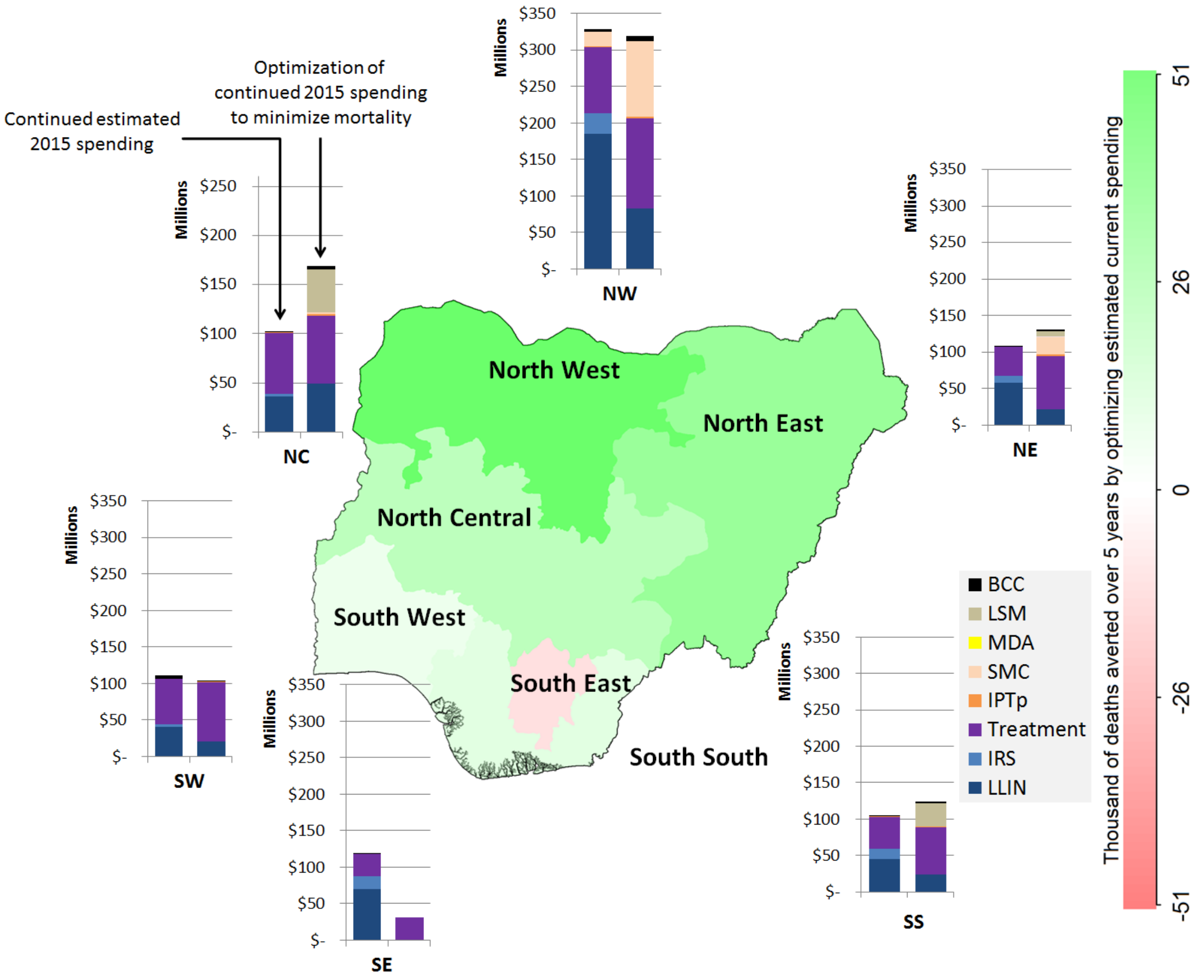

Figure D10: Geospatial optimization to minimize mortality. Geospatially optimized 5-year spending of estimated current budget compared to current allocations. Abbreviations: BCC, behavioural change communication; IPTp, intermittent presumptive treatment during pregnancy; IRS, indoor residual spraying; LLINs, long lasting insecticide treated nets; LSM, larval source management; MDA, mass drug administration; SMC, seasonal mass chemoprevention in children.

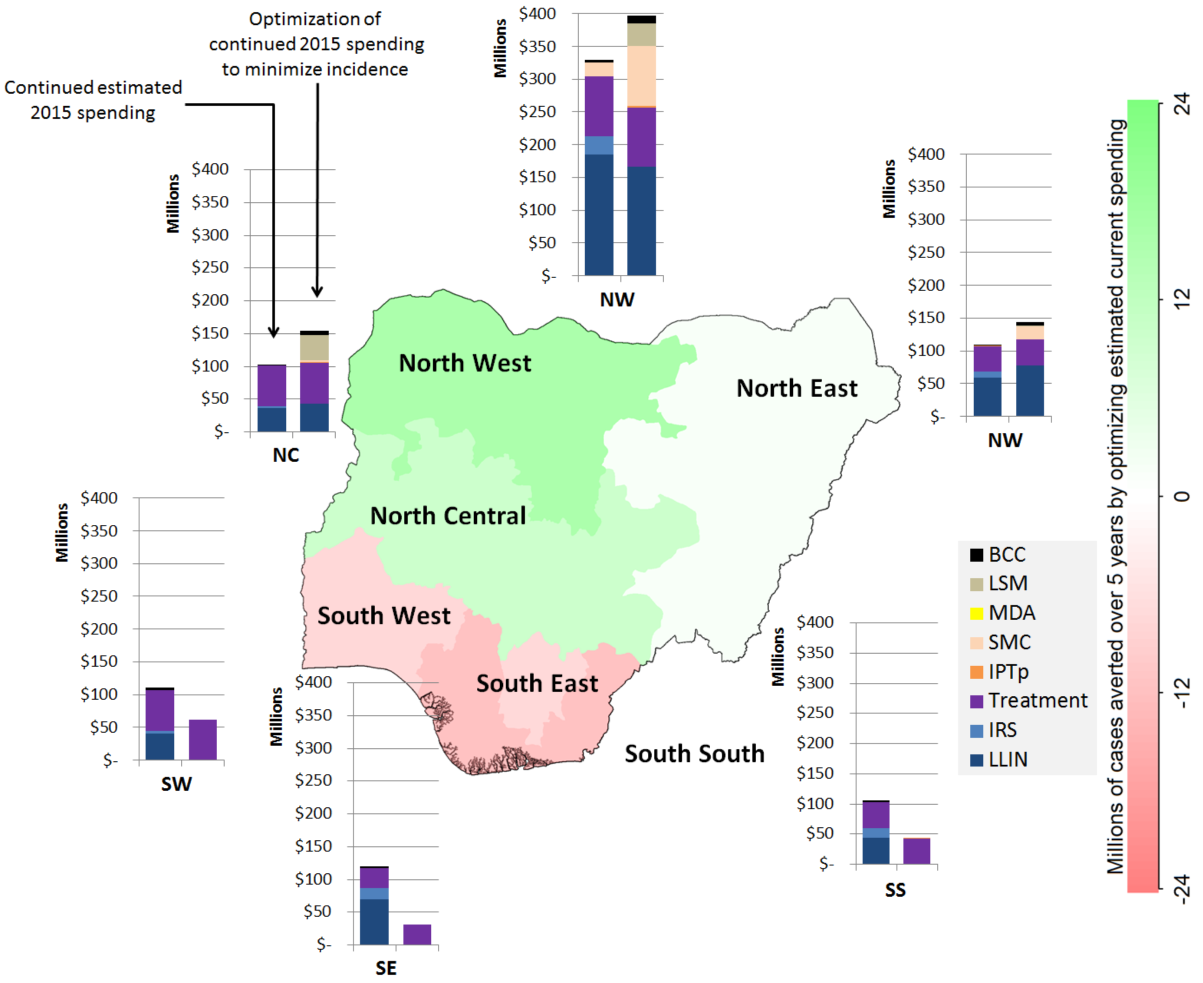

Figure D11: Geospatial optimization to minimize incidence. Geospatially optimized 5-year spending of estimated current budget compared to current allocations. Abbreviations: BCC, behavioural change communication; IPTp, intermittent presumptive treatment during pregnancy; IRS, indoor residual spraying; LLINs, long lasting insecticide treated nets; LSM, larval source management; MDA, mass drug administration; SMC, seasonal mass chemoprevention in children.

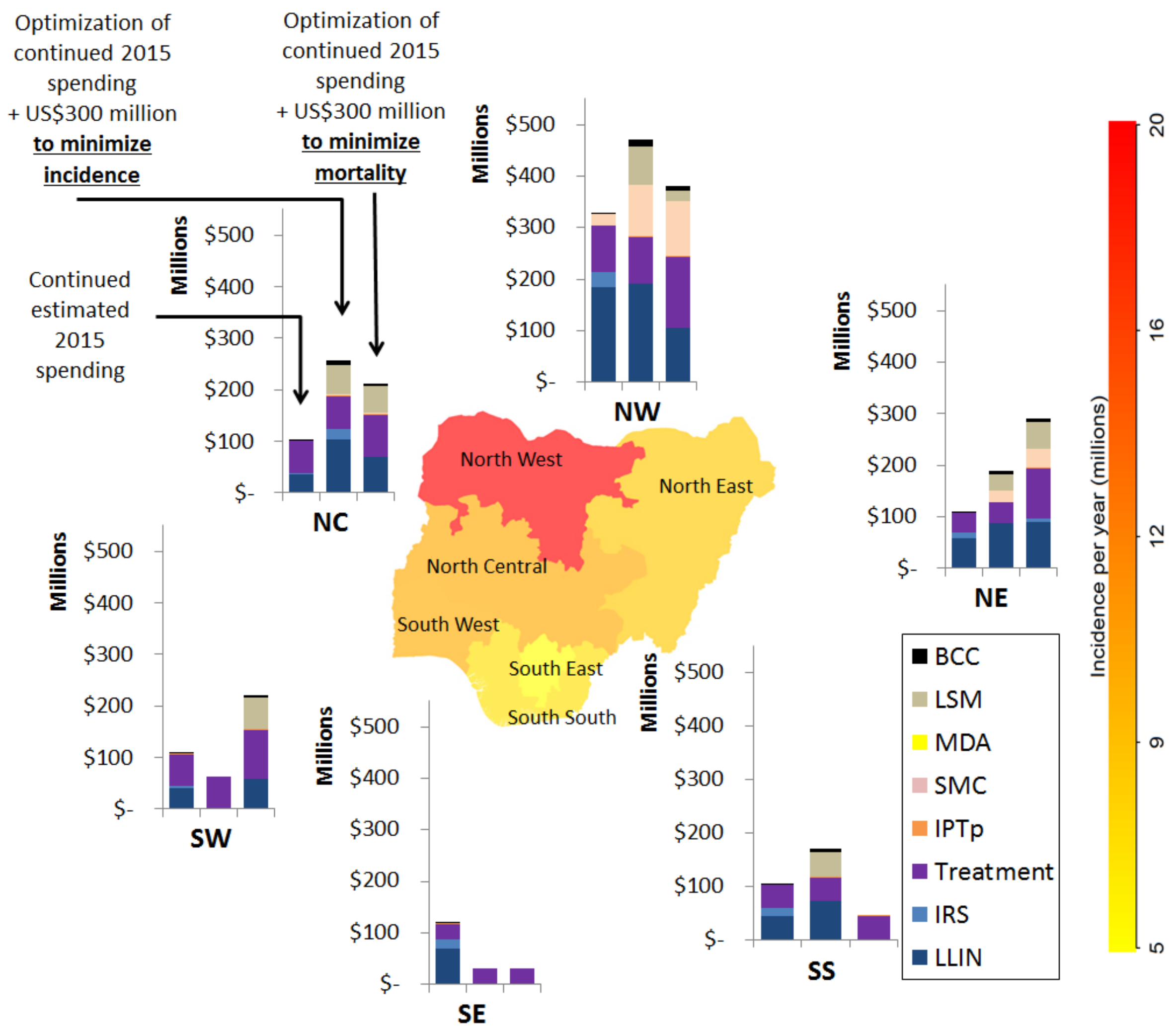

Figure D12: Comparison of geospatial optimizations to minimize mortality and incidence. Geospatially optimized 5-year spending of estimated current budget + US$300 million, compared to current allocations. Abbreviations: BCC, behavioural change communication; IPTp, intermittent presumptive treatment during pregnancy; IRS, indoor residual spraying; LLINs, long lasting insecticide treated nets; LSM, larval source management; MDA, mass drug administration; SMC, seasonal mass chemoprevention in children.

References

1. United Nations Department of Economic and Social Affairs Population Division: **World Population Prospects: The 2015 Revision, DVD Edition**. 2015.
2. UN Population Division: http://www.un.org/en/development/desa/population/. 2016.
3. Idris B, Isah B, Chukwuemeka V, Ukubuiwe A: **Seasonal trends in epidemiological and entomological profiles of malaria transmission in North Central Nigeria**. *Pakistan Journal of Biological Sciences* 2011, **14**(4):293-299.
4. Malaria Consortium: **Seasonal Malaria Chemoprovention. Available from: http://www.malariaconsortium.org/media-downloads/197/Seasonal%20Malaria%20Chemoprevention%20(SMC)%20in%20Nigeria**. 2013.
5. Koella J, Antia R: **Epidemiological models for the spread of anti-malarial resistance**. *Malaria Journal* 2003, **2**(1):3.
6. Aron JL, May RM: **The population dynamics of malaria**. In: *The population dynamics of infectious diseases: theory and applications.* edn.: Springer; 1982: 139-179.
7. Mandal S, Sarkar RR, Sinha S: **Mathematical models of malaria-a review**. *Malar J* 2011, **10**(202):10.1186.
8. Wise Solutions LLC, Center for Communications Programs-Nigeria: **Faiths United for Health Campaign, Evaluation Report. Available from http://dmeforpeace.org/sites/default/files/CIFA%20NIFAA%20Program%20Evaluation%202011.pdf**. 2011.
9. Kerr C, Smolinski T, Dura-Bernal S, Wilson D: **Optimization by Bayesian adaptive locally linear stochastic descent**. *Nature Scientific Reports* under review.
10. Churcher TS, Trape JF, Cohuet A: **Human-to-mosquito transmission efficiency increases as malaria is controlled**. *Nat Commun* 2015, **6**:6054.
11. Anderson RM, May RM, Anderson B: **Infectious diseases of humans: dynamics and control**, vol. 28: Wiley Online Library; 1992.
12. Labadin J, Kon C, Juan S: **Deterministic malaria transmission model with acquired immunity**. In: *Proceedings of the World Congress on Engineering and Computer Science: 2009*; 2009: 20-22.
13. Chitnis N, Cushing J, Hyman J: **Bifurcation analysis of a mathematical model for malaria transmission**. *SIAM Journal on Applied Mathematics* 2006, **67**(1):24-45.
14. Baton LA, Ranford-Cartwright LC: **Spreading the seeds of million-murdering death: metamorphoses of malaria in the mosquito**. *Trends in parasitology* 2005, **21**(12):573-580.
15. Mehlhorn H, Armstrong PM: **Encyclopedic reference of parasitology: Diseases, treatment, therapy**, vol. 2: Springer Science & Business Media; 2001.
16. Dietz K, Molineaux L, Thomas A: **A malaria model tested in the African savannah**. *Bulletin of the World Health Organization* 1974, **50**(3-4):347.
17. Chitnis N, Hyman JM, Cushing JM: **Determining important parameters in the spread of malaria through the sensitivity analysis of a mathematical model**. *Bulletin of mathematical biology* 2008, **70**(5):1272-1296.
18. Vaughan JA: **Population dynamics of Plasmodium sporogony**. *Trends in parasitology* 2007, **23**(2):63-70.
19. Walker PG, Griffin JT, Ferguson NM, Ghani AC: **Estimating the most efficient allocation of interventions to achieve reductions in Plasmodium falciparum malaria burden and transmission in Africa: a modelling study**. *The Lancet Global Health* 2016.
20. Gamble C, Ekwaru PJ, Garner P, Ter Kuile FO: **Insecticide-treated nets for the prevention of malaria in pregnancy: a systematic review of randomised controlled trials**. *PLoS Med* 2007, **4**(3):e107.
21. Lengeler C: **Insecticide-treated bed nets and curtains for preventing malaria**. *Cochrane Database Syst Rev* 2004, **2**(2).

22. Griffin JT, Hollingsworth TD, Okell LC, Churcher TS, White M, Hinsley W, Bousema T, Drakeley CJ, Ferguson NM, Basáñez M-G: **Reducing Plasmodium falciparum malaria transmission in Africa: a model-based evaluation of intervention strategies**. *PLoS Med* 2010, **7**(8):e1000324.
23. Pluess B, Tanser FC, Lengeler C, Sharp BL: **Indoor residual spraying for preventing malaria**. *Cochrane Database Syst Rev* 2010, **4**(4).
24. Okell LC, Cairns M, Griffin JT, Ferguson NM, Tarning J, Jagoe G, Hugo P, Baker M, D'Alessandro U, Bousema T: **Contrasting benefits of different artemisinin combination therapies as first-line malaria treatments using model-based cost-effectiveness analysis**. *Nature communications* 2014, **5**.
25. SuNMaP: **Malaria Control State Fact Sheets. Available from http://www.malariaconsortium.org/media-downloads/684/Malaria%20control%20Nigeria:%20state%20fact%20sheets**. 2015.
26. Meremikwu MM, Donegan S, Esu E: **Chemoprophylaxis and intermittent treatment for preventing malaria in children**. *The Cochrane Library* 2008.
27. Wilson AL: **A systematic review and meta-analysis of the efficacy and safety of intermittent preventive treatment of malaria in children (IPTc)**. *PloS one* 2011, **6**(2):e16976.
28. Meremikwu MM, Donegan S, Sinclair D, Esu E, Oringanje C: **Intermittent preventive treatment for malaria in children living in areas with seasonal transmission**. *Cochrane Database Syst Rev* 2012, **2**(2).
29. Tusting LS, Thwing J, Sinclair D, Fillinger U, Gimnig J, Bonner KE, Bottomley C, Lindsay SW: **Mosquito larval source management for controlling malaria**. *The Cochrane Library* 2013.
30. Department for International Development Nigeria: **Costed extension of the DFID Support to National Malaria Programme (SUNMAP), Business Case**. 2013.
31. Hansen KS, Grieve E, Mikhail A, Mayan I, Mohammed N, Anwar M, Baktash SH, Drake TL, Whitty CJ, Rowland MW: **Cost-effectiveness of malaria diagnosis using rapid diagnostic tests compared to microscopy or clinical symptoms alone in Afghanistan**. *Malaria journal* 2015, **14**(1):1-15.
32. National Population Commission (NCP), National Malaria Control Programme (NMCP), International. I: **Nigeria Malaria Indicator Survey 2010, Final Report**. *Abuja, Nigeria: NPC, NMCP, and ICF International* 2012.
33. World Health Organization: **WHO recommendations for achieving universal coverage with long-lasting insecticidal nets in malaria control. Available from http://www.who.int/malaria/publications/atoz/who_recommendations_universal_coverage_llins.pdf**. 2014.
34. **IRS Quantification and Budget 2013-2016 Final**. 2016.
35. The World Bank: **Official exchange rate. Available from http://data.worldbank.org/indicator/PA.NUS.FCRF**. 2016.
36. President's Malaria Initiative: **Nigeria; Malaria Operational Plan FY 2015. Available from https://www.pmi.gov/docs/default-source/default-document-library/malaria-operational-plans/fy-15/fy-2015-nigeria-malaria-operational-plan.pdf?sfvrsn=6**. 2015.
37. White MT, Conteh L, Cibulskis R, Ghani AC: **Costs and cost-effectiveness of malaria control interventions-a systematic review**. *Malar J* 2011, **10**(337):1475-2875.
38. Worrall E, Fillinger U: **Large-scale use of mosquito larval source management for malaria control in Africa: a cost analysis**. *Malaria journal* 2011, **10**(1):1.
39. Hay SI, Snow RW: **The Malaria Atlas Project: developing global maps of malaria risk**. *PLoS Med* 2006, **3**(12):e473.
40. Malaria Altas Project: http://www.map.ox.ac.uk/. 2016.
41. Index mundi: **Nigerian birth rate estimate. http://www.indexmundi.com/g/g.aspx?c=ni&v=25**. 2014.

42. Cameron E, Battle KE, Bhatt S, Weiss DJ, Bisanzio D, Mappin B, Dalrymple U, Hay SI, Smith DL, Griffin JT: **Defining the relationship between infection prevalence and clinical incidence of Plasmodium falciparum malaria**. *Nature communications* 2015, **6**.
43. van Eijk AM, Hill J, Noor AM, Snow RW, ter Kuile FO: **Prevalence of malaria infection in pregnant women compared with children for tracking malaria transmission in sub-Saharan Africa: a systematic review and meta-analysis**. *The Lancet Global Health* 2015, **3**(10):e617-e628.
44. World Health Organization: **World Malaria Report. Available from http://apps.who.int/iris/bitstream/10665/200018/1/9789241565158_eng.pdf?ua=1**. 2015.
45. Malaria Atlas Project: **http://www.map.ox.ac.uk/**. 2016.
